# Interplay between BRCA1 and RHAMM Regulates Epithelial Apicobasal Polarization and May Influence Risk of Breast Cancer

**DOI:** 10.1371/journal.pbio.1001199

**Published:** 2011-11-15

**Authors:** Christopher A. Maxwell, Javier Benítez, Laia Gómez-Baldó, Ana Osorio, Núria Bonifaci, Ricardo Fernández-Ramires, Sylvain V. Costes, Elisabet Guinó, Helen Chen, Gareth J. R. Evans, Pooja Mohan, Isabel Català, Anna Petit, Helena Aguilar, Alberto Villanueva, Alvaro Aytes, Jordi Serra-Musach, Gad Rennert, Flavio Lejbkowicz, Paolo Peterlongo, Siranoush Manoukian, Bernard Peissel, Carla B. Ripamonti, Bernardo Bonanni, Alessandra Viel, Anna Allavena, Loris Bernard, Paolo Radice, Eitan Friedman, Bella Kaufman, Yael Laitman, Maya Dubrovsky, Roni Milgrom, Anna Jakubowska, Cezary Cybulski, Bohdan Gorski, Katarzyna Jaworska, Katarzyna Durda, Grzegorz Sukiennicki, Jan Lubiński, Yin Yao Shugart, Susan M. Domchek, Richard Letrero, Barbara L. Weber, Frans B. L. Hogervorst, Matti A. Rookus, J. Margriet Collee, Peter Devilee, Marjolijn J. Ligtenberg, Rob B. van der Luijt, Cora M. Aalfs, Quinten Waisfisz, Juul Wijnen, Cornelis E. P. van Roozendaal, HEBON, EMBRACE, Douglas F. Easton, Susan Peock, Margaret Cook, Clare Oliver, Debra Frost, Patricia Harrington, D. Gareth Evans, Fiona Lalloo, Rosalind Eeles, Louise Izatt, Carol Chu, Diana Eccles, Fiona Douglas, Carole Brewer, Heli Nevanlinna, Tuomas Heikkinen, Fergus J. Couch, Noralane M. Lindor, Xianshu Wang, Andrew K. Godwin, Maria A. Caligo, Grazia Lombardi, Niklas Loman, Per Karlsson, Hans Ehrencrona, Anna von Wachenfeldt, SWE-BRCA, Rosa Bjork Barkardottir, Ute Hamann, Muhammad U. Rashid, Adriana Lasa, Trinidad Caldés, Raquel Andrés, Michael Schmitt, Volker Assmann, Kristen Stevens, Kenneth Offit, João Curado, Hagen Tilgner, Roderic Guigó, Gemma Aiza, Joan Brunet, Joan Castellsagué, Griselda Martrat, Ander Urruticoechea, Ignacio Blanco, Laima Tihomirova, David E. Goldgar, Saundra Buys, Esther M. John, Alexander Miron, Melissa Southey, Mary B. Daly, BCFR, Rita K. Schmutzler, Barbara Wappenschmidt, Alfons Meindl, Norbert Arnold, Helmut Deissler, Raymonda Varon-Mateeva, Christian Sutter, Dieter Niederacher, Evgeny Imyamitov, Olga M. Sinilnikova, Dominique Stoppa-Lyonne, Sylvie Mazoyer, Carole Verny-Pierre, Laurent Castera, Antoine de Pauw, Yves-Jean Bignon, Nancy Uhrhammer, Jean-Philippe Peyrat, Philippe Vennin, Sandra Fert Ferrer, Marie-Agnès Collonge-Rame, Isabelle Mortemousque, GEMO Study Collaborators, Amanda B. Spurdle, Jonathan Beesley, Xiaoqing Chen, Sue Healey, kConFab, Mary Helen Barcellos-Hoff, Marc Vidal, Stephen B. Gruber, Conxi Lázaro, Gabriel Capellá, Lesley McGuffog, Katherine L. Nathanson, Antonis C. Antoniou, Georgia Chenevix-Trench, Markus C. Fleisch, Víctor Moreno, Miguel Angel Pujana

**Affiliations:** 1 Translational Research Laboratory, Catalan Institute of Oncology, Bellvitge Biomedical Research Institute (IDIBELL), L'Hospitalet, Catalonia, Spain; 2 Human Cancer Genetics Programme, Spanish National Cancer Research Centre, Madrid, Spain; 3 Biomedical Research Centre Network for Rare Diseases, Spain; 4 Biomedical Research Centre Network for Epidemiology and Public Health, Spain; 5 Biomarkers and Susceptibility Unit, Catalan Institute of Oncology, IDIBELL, L'Hospitalet, Catalonia, Spain; 6 Life Sciences Division, Lawrence Berkeley National Laboratory, Berkeley, California, United States of America; 7 Child and Family Research Institute, Vancouver, British Columbia, Canada; 8 Department of Pathology, University Hospital of Bellvitge, IDIBELL, L'Hospitalet, Catalonia, Spain; 9 CHS National Cancer Control Center, Department of Community Medicine and Epidemiology, Carmel Medical Center and B. Rappaport Faculty of Medicine, Technion, Haifa, Israel; 10 Unit of Molecular Bases of Genetic Risk and Genetic Testing, Department of Preventive and Predictive Medicine, Fondazione IRCCS Istituto Nazionale Tumori, and IFOM Fondazione Istituto FIRC di Oncologia Molecolare, Milan, Italy; 11 Unit of Medical Genetics, Department of Preventive and Predictive Medicine, Fondazione IRCCS Istituto Nazionale Tumori, Milan, Italy; 12 Division of Cancer Prevention and Genetics, Istituto Europeo di Oncologia, Milan, Italy; 13 Division of Experimental Oncology 1, Centro di Riferimento Oncologico, IRCCS, Aviano, Italy; 14 Department of Genetics, Biology and Biochemistry, University of Turin, Turin, Italy; 15 Department of Experimental Oncology, Istituto Europeo di Oncologia, and Consortium for Genomics Technology (Cogentech), Milan, Italy; 16 The Susanne Levy Gertner Oncogenetics Unit, Institute of Human Genetics, Chaim Sheba Medical Center, Ramat Gan, Israel; 17 Sackler Faculty of Medicine, Tel Aviv University, Ramat Aviv, Israel; 18 International Hereditary Cancer Centre, Department of Genetics and Pathology, Pomeranian Medical University, Szczecin, Poland; 19 Unit of Statistical Genetics, Division of Intramural Research Program, National Institute of Mental Health, National Institute of Health, Bethesda, Maryland, United States of America; 20 Abramson Cancer Center, University of Pennsylvania School of Medicine, Philadelphia, Pennsylvania, United States of America; 21 Family Cancer Clinic, Department of Pathology, The Netherlands Cancer Institute, Amsterdam, the Netherlands; 22 Department of Epidemiology, The Netherlands Cancer Institute, Amsterdam, the Netherlands; 23 Department of Clinical Genetics, Rotterdam Family Cancer Clinic, Erasmus University Medical Center, Rotterdam, the Netherlands; 24 Department of Genetic Epidemiology, Leiden University Medical Center, Leiden, the Netherlands; 25 Department of Human Genetics, Radboud University Medical Center, Nijmegen, the Netherlands; 26 Department of Clinical Molecular Genetics, Utrecht University Medical Center, Utrecht, the Netherlands; 27 Department of Clinical Genetics, Academic Medical Center, Amsterdam, the Netherlands; 28 Department of Clinical Genetics, VU University Medical Center, Amsterdam, the Netherlands; 29 Center for Human and Clinical Genetics, Leiden University Medical Center, Leiden, the Netherlands; 30 Department of Clinical Genetics, University Medical Center, Maastricht, the Netherlands; 31 Hereditary Breast and Ovarian Cancer Group, the Netherlands; 32 Centre for Cancer Genetic Epidemiology, Department of Public Health and Primary Care, University of Cambridge, Cambridge, United Kingdom; 33 Department of Oncology, University of Cambridge, Cambridge, United Kingdom; 34 Genetic Medicine, Manchester Academic Health Sciences Centre, Central Manchester University Hospitals NHS Foundation Trust, Manchester, United Kingdom; 35 The Oncogenetics Team, The Institute of Cancer Research and Royal Marsden NHS Foundation Trust, Surrey, United Kingdom; 36 Clinical Genetics, Guy's and St. Thomas' NHS Foundation Trust, London, United Kingdom; 37 Yorkshire Regional Genetics Service, St. James's Hospital, Leeds, United Kingdom; 38 Wessex Clinical Genetics Service, Princess Anne Hospital, Southampton, United Kingdom; 39 Institute of Human Genetics, Centre for Life, Newcastle Upon Tyne Hospitals NHS Trust, Newcastle upon Tyne, United Kingdom; 40 Department of Clinical Genetics, Royal Devon & Exeter Hospital, Exeter, United Kingdom; 41 Department of Obstetrics and Gynecology, Helsinki University Central Hospital, Helsinki, Finland; 42 Department of Laboratory Medicine and Pathology, Mayo Clinic, Rochester, Minnesota, United States of America; 43 Department of Medical Genetics, Mayo Clinic, Rochester, Minnesota, United States of America; 44 Department of Pathology and Laboratory Medicine, University of Kansas Medical Center, Kansas City, Kansas, United States of America; 45 Section of Genetic Oncology, Department of Oncology, University of Pisa, and Department of Laboratory Medicine, University Hospital of Pisa, Pisa, Italy; 46 Department of Oncology, Lund University Hospital, Lund, Sweden; 47 Department of Oncology, Sahlgrenska University Hospital, Gothenburg, Sweden; 48 Department of Genetics and Pathology, Rudbeck Laboratory, Uppsala University, Uppsala, Sweden; 49 Department of Oncology, Karolinska University Hospital, Stockholm, Sweden; 50 Swedish Breast Cancer Study, Sweden; 51 Department of Pathology, Landspitali-University Hospital, Reykjavik, Iceland; 52 Molecular Genetics of Breast Cancer, Deutsches Krebsforschungszentrum, Heidelberg, Germany; 53 Molecular Genetics of Breast Cancer, Deutsches Krebsforschungszentrum, Heidelberg, Germany, and Department of Basic Sciences, Shaukat Khanum Memorial Cancer Hospital and Research Centre, Lahore, Pakistan; 54 Genetic Service, Hospital de la Santa Creu i Sant Pau, Barcelona, Catalonia, Spain; 55 Molecular Oncology Laboratory, Hospital Clínico San Carlos, Madrid, Spain; 56 Medical Oncology Division, Hospital Clínico de Zaragoza, Zaragoza, Spain; 57 Department of Internal Medicine III, University of Rostock, Rostock, Germany; 58 Center for Experimental Medicine, Institute of Tumor Biology, University Hospital Hamburg–Eppendorf, Hamburg, Germany; 59 Department of Epidemiology, University of Michigan, Ann Arbor, Michigan, United States of America; 60 Clinical Genetics Service, Department of Medicine, Memorial Sloan-Kettering Cancer Center, New York, New York, United States of America; 61 Bioinformatics and Genomics Group, Centre for Genomic Regulation (CRG), Biomedical Research Park of Barcelona (PRBB), Barcelona, Catalonia, Spain; 62 Genetic Counseling and Hereditary Cancer Programme, Catalan Institute of Oncology, IDIBELL and Girona Biomedical Research Institute (IdIBGi), Catalonia, Spain; 63 Latvian Biomedical Research and Study Center, Riga, Latvia; 64 Department of Dermatology, University of Utah School of Medicine, Salt Lake City, Utah, United States of America; 65 Department of Internal Medicine, Huntsman Cancer Institute, Salt Lake City, Utah, United States of America; 66 Cancer Prevention Institute of California, Fremont, California, United States of America; 67 Department of Cancer Biology, Dana-Farber Cancer Institute, and Department of Surgery, Harvard Medical School, Boston, Massachusetts, United States of America; 68 Centre for Molecular, Environmental, Genetic and Analytic (MEGA) Epidemiology, Melbourne School of Population Health, The University of Melbourne, Victoria, Australia; 69 Division of Population Science, Fox Chase Cancer Center, Philadelphia, Pennsylvania, United States of America; 70 Breast Cancer Family Registry, United States of America; 71 Center for Familial Breast and Ovarian Cancer and Center of Integrated Oncology, University of Cologne, Cologne, Germany; 72 Department of Obstetrics and Gynaecology, Klinikum rechts der Isar, Technical University, Munich, Germany; 73 Division of Oncology, Department of Gynaecology and Obstetrics, University Hospital Schleswig-Holstein, Kiel, Germany; 74 Department of Obstetrics and Gynecology, Ulm University, Ulm, Germany; 75 Institut für Humangenetik, Charité-Universitätsmedizin Berlin, Berlin, Germany; 76 Institute of Human Genetics, University of Heidelberg, Heidelberg, Germany; 77 Division of Molecular Genetics, Department of Gynaecology and Obstetrics, Clinical Center University of Düsseldorf, Düsseldorf, Germany; 78 N. N. Petrov Institute of Oncology, Saint-Petersburg, Russian Federation; 79 Unité Mixte de Génétique Constitutionnelle des Cancers Fréquents, Centre Hospitalier Universitaire de Lyon, Centre Léon Bérard, Lyon, France; 80 Equipe labellisée LIGUE 2008, UMR5201 CNRS, Centre Léon Bérard, Université de Lyon, Lyon, France; 81 INSERM U509, Service de Génétique Oncologique, Institut Curie, Université Paris-Descartes, Paris, France; 82 Département d'Oncogénétique, Centre Jean Perrin, Université de Clermont-Ferrand, Clermont-Ferrand, France; 83 Laboratoire d'Oncologie Moléculaire Humaine, Centre Oscar Lambret, Lille, France; 84 Consultation d'Oncogénétique, Centre Oscar Lambret, Lille, France; 85 Laboratoire de Génétique Chromosomique, Hôtel Dieu Centre Hospitalier, Chambéry, France; 86 Service de Génétique-Histologie-Biologie du Développement et de la Reproduction, Centre Hospitalier Universitaire de Besançon, Besançon, France; 87 Service de Génétique, Centre Hospitalier Universitaire Bretonneau, Tours, France; 88 GEMO Study (Genetics Network “Groupe Génétique et Cancer”), Fédération Nationale des Centres de Lutte Contre le Cancer, France; 89 Queensland Institute of Medical Research, Brisbane, Australia; 90 The Kathleen Cuningham Foundation Consortium for Research into Familial Breast Cancer, Peter MacCallum Cancer Institute, East Melbourne, Australia; 91 Center for Cancer Systems Biology (CCSB) and Department of Cancer Biology, Dana-Farber Cancer Institute, and Department of Genetics, Harvard Medical School, Boston, Massachusetts, United States of America; 92 Department of Internal Medicine, Epidemiology, Human Genetics, University of Michigan, Ann Arbor, Michigan, United States of America; 93 Department of Obstetrics and Gynaecologie, Heinrich-Heine-University, Duesseldorf, Germany; Breakthrough Breast Cancer Research Center, United Kingdom

## Abstract

Differentiated mammary epithelium shows apicobasal polarity, and loss of tissue organization is an early hallmark of breast carcinogenesis. In *BRCA1* mutation carriers, accumulation of stem and progenitor cells in normal breast tissue and increased risk of developing tumors of basal-like type suggest that BRCA1 regulates stem/progenitor cell proliferation and differentiation. However, the function of BRCA1 in this process and its link to carcinogenesis remain unknown. Here we depict a molecular mechanism involving BRCA1 and RHAMM that regulates apicobasal polarity and, when perturbed, may increase risk of breast cancer. Starting from complementary genetic analyses across families and populations, we identified common genetic variation at the low-penetrance susceptibility *HMMR* locus (encoding for RHAMM) that modifies breast cancer risk among *BRCA1*, but probably not *BRCA2*, mutation carriers: *n* = 7,584, weighted hazard ratio (_w_HR) = 1.09 (95% CI 1.02–1.16), *p_trend_* = 0.017; and *n* = 3,965, _w_HR = 1.04 (95% CI 0.94–1.16), *p_trend_* = 0.43; respectively. Subsequently, studies of MCF10A apicobasal polarization revealed a central role for BRCA1 and RHAMM, together with AURKA and TPX2, in essential reorganization of microtubules. Mechanistically, reorganization is facilitated by BRCA1 and impaired by AURKA, which is regulated by negative feedback involving RHAMM and TPX2. Taken together, our data provide fundamental insight into apicobasal polarization through BRCA1 function, which may explain the expanded cell subsets and characteristic tumor type accompanying *BRCA1* mutation, while also linking this process to sporadic breast cancer through perturbation of *HMMR*/RHAMM.

## Abstract 2

Genetic analysis identifies the HMMR gene as a modifier of the breast cancer risk associated with BRCA1 gene mutation, while cell biological analysis of the protein product suggests a function in regulating development of the mammary gland.

## Author Summary

Mutations in two genes that were initially identified as predisposing carriers to early-onset breast cancer, *BRCA1* and *BRCA2*, cause similar perturbations in cellular responses to DNA damage but predispose carriers to distinct tumor types. Thus, the two genes may trigger different carcinogenic processes. We have used genetic analyses of affected families to uncover additional genetic variation that is linked to the risk of developing cancer for carriers of *BRCA1* mutations. This variation falls within a centrosomal gene, named *HMMR*. The protein product of *HMMR*, which is called RHAMM, works in concert with BRCA1 to regulate the structure of normal breast cells as they grow and become polarized. This polarization process depends upon a balance between the activities of BRCA1 and the Aurora kinase A, with the kinase opposing BRCA1 function and promoting growth. Our findings provide new insights into the mechanism through which BRCA1 may promote commitment of initially bipotent mammary cells towards the luminal lineage, and how loss of this function may predispose cells to become breast tumors of a basal-like type.

## Introduction

The mammary gland is composed of two epithelial cell lineages that form an inner apicobasal-polarized luminal layer surrounded by an outer, or basal, layer of contractile myoepithelial cells [1]. Epithelial cell subsets are likely maintained through a differentiation hierarchy supported by an estrogen receptor (ER)-negative mammary stem cell population enriched at the basal compartment [2]–[7]. Cytoskeletal structures, including actin and intermediate filament content, identify differentiated cells [8] and may therefore contribute to differentiation. For example, the organization of microtubules at adherens junctions is essential for the maintenance of cell-to-cell contacts in apicobasal-polarized epithelial [9]. This involves centrosome-dependent microtubule assembly followed by release and capture at non-centrosome sites [10]. Therefore, dynamic cytoskeletal reorganization may be critical to the terminal differentiation of breast luminal epithelium. However, the molecular determinants of this process and the link with carcinogenesis remain unknown.

The common pathological features of breast tumors arising in *breast cancer 1, early onset* (*BRCA1*) gene mutation carriers, including the basal-like phenotype and ER negativity [11],[12], led to the proposition that BRCA1 function regulates stem/progenitor cell proliferation and differentiation [13]. Recent evidence supports this hypothesis. Cell proliferation and differentiation are altered with BRCA1 depletion in the non-tumorigenic MCF10A breast cell line [14] and with ex vivo culture of primary mammary epithelial cells from *BRCA1* mutation carriers [15]. Xenografts of primary mammary epithelial cells depleted of BRCA1 show expansion of stem cells with impaired luminal differentiation [16]. Expanded luminal progenitor populations have also been detected in breast tissue from *BRCA1* mutation carriers [17] and, subsequently, proposed as the target of transformation leading to basal-like tumors [18]. A more recent study has shown expanded basal progenitor cells but also defects in luminal progenitor differentiation in these carriers [19]. While it has been postulated that stem/progenitor cells may have stringent requirements for high-fidelity DNA damage repair [17], the potential contribution of BRCA1 to other molecular events fundamental in differentiation remains to be elucidated.

BRCA1-dependent ubiquitination, functioning as a heterodimer with BRCA1-associated RING domain 1 (BARD1), down-regulates assembly of centrosome microtubules in a mammary-specific manner [20],[21]. *Xenopus* brca1-bard1 attenuates the function of a microtubule-associated protein called *Xenopus* receptor for hyaluronan-mediated motility (xrhamm) [22]. Xrhamm is the ortholog of a candidate low-penetrance breast cancer susceptibility gene product (RHAMM, *HMMR* gene) [23] whose over-expression in tumors is associated with poor prognosis and early age at diagnosis [23]–[25]. While xrhamm regulates microtubule organization during meiosis [26], RHAMM controls γ-tubulin (TUBG1) recruitment [27] and interphase microtubule dynamics [28]. Together, these observations suggest that BRCA1 might be involved in epithelial differentiation by down-regulating centrosome microtubule assembly, through RHAMM and TUBG1, and promoting the cytoskeletal reorganization necessary for apicobasal polarization. Conversely, loss of BRCA1 function might impair structural cues of terminal differentiation and, consequently, increase risk of breast cancer characterized by the basal-like tumor type. Here, we conduct complementary analyses to demonstrate genetic, molecular, and functional interactions between *BRCA1*/BRCA1, *HMMR*/RHAMM, and additional centrosome components that orchestrate cytoskeletal reorganization critical for epithelial apicobasal polarization. These new insights may enhance our understanding of mammary epithelial differentiation and the link with breast carcinogenesis.

## Results

### Common Genetic Variation in *HMMR* Modifies Breast Cancer Risk among *BRCA1* Mutation Carriers

Although BRCA1 and BRCA2 function coordinately during DNA damage response, genomic, transcriptomic, molecular, and pathological features of breast tumors arising in *BRCA1* and *BRCA2* mutation carriers suggest that carcinogenesis may occur through perturbation of shared and distinct biological processes [13],[29]. Previous analysis of candidate genomic regions using a linkage approach suggested specific modification of breast cancer risk among *BRCA1* mutation carriers by common genetic variation at chromosome 5q33-34 [30]. Extension of this study supports the original conclusion: a haplotype analysis in 27 families with *BRCA1* mutations revealed a nonparametric linkage score peak of 4.24 at the 5q34 region containing *HMMR* (Table S1); in contrast, no evidence of linkage was observed among 16 families with *BRCA2* mutations (only a suggestive signal at 20 centiMorgans distal of *HMMR* was detected, *D5S408* nonparametric linkage score = 1.91).

**Figure 1:**
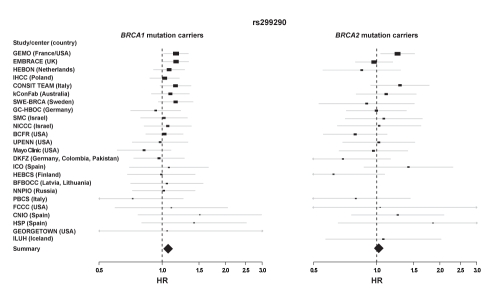
Effect of *HMMR* rs299290 variation on breast cancer risk among *BRCA1* and *BRCA2* mutation carriers. Forrest plots show HRs and 95% CIs of the additive model (rs299290 C allele) for all participating centers ordered by sample size (*n*>30) of *BRCA1* mutation carriers (left panel, _w_HR per study center are shown in Table S2; right panel, effect on *BRCA2* mutation carriers). The size of the rectangles is proportional to the corresponding study precision.

Common breast cancer-predisposition alleles may differentially modify breast cancer risk among *BRCA1* and *BRCA2* mutation carriers [31]–[33]. To complement the linkage approach, we evaluated the effect of common *HMMR* genetic variation [23] on breast cancer risk in *BRCA1* and *BRCA2* mutation carriers. Following a pilot study in Italy and Spain, analysis of carriers (*n* = 11,609) collected through 24 study groups participating in the Consortium of Investigators of Modifiers of *BRCA1/2* (CIMBA) detected significant modification of breast cancer risk by *HMMR* rs299290 variant among *BRCA1*, but not *BRCA2*, mutation carriers: *BRCA1* mutation carriers *n* = 7,584, Cox proportional-hazards regression model, hazard ratio (HR) = 1.08 (95% confidence interval (CI) 1.02–1.13), *p_trend_* = 0.004 (*p*_2df_ = 0.014), in the same direction as originally detected in Ashkenazi Jewish populations [23]; *BRCA2* mutation carriers *n* = 3,965, HR = 1.03 (95% CI 0.96–1.10), *p_trend_* = 0.42 (*p*_2df_ = 0.67). For *BRCA1* mutation carriers, consistent effects were observed across centers with larger sample sizes (Figure 1).

We performed a number of sensitivity analyses to investigate the robustness of our results. First, since prophylactic oophorectomy reduces the risk of breast cancer in *BRCA1* mutation carriers by up to 50% [34], we included this observation as a time-dependent covariate in the analysis, and a significant association similar to the one shown above was revealed: HR = 1.09 (95% CI 1.03–1.16), *p_trend_* = 4.5×10^−4^. Second, a non-significant association, but in the same direction, was identified when prevalent cases (defined as those diagnosed with breast cancer more than five years before recruitment) were excluded from the analysis: HR = 1.06 (95% CI 0.99–1.15), *p_trend_* = 0.10. Finally, to investigate whether the retrospective study design and the non-random sampling of affected and unaffected mutation carriers introduce bias into the HR estimates, the data were also analyzed using a weighted cohort approach [35], which yielded similar results to those shown above: *BRCA1* mutation carriers _w_HR = 1.09 (95% CI 1.02–1.16), *p_trend_* = 0.017 (*p*_2df_ = 0.041) (_w_HR per study centre are detailed in Table S2); *BRCA2* mutation carriers _w_HR = 1.04 (95% CI 0.94–1.16), *p_trend_* = 0.43 (*p*_2df_ = 0.68). Examination of heterogeneity in risk estimates across groups did not show significant differences under the multiplicative model (*p_het_*≥0.3). The association was then evaluated according to the predicted functional consequences of *BRCA1* mutation type [36]–[40]. This analysis suggested an effect in carriers of loss-of-function mutations expected to result in a reduced transcript or protein level due to nonsense-mediated RNA decay (*n* = 4,636, _w_HR = 1.08 (95% CI 0.99–1.19)), whereas carriers of mutations likely to generate stable proteins with potential residual or dominant negative function might not be influenced (*n* = 1,380, _w_HR = 1.00 (95% CI 0.85–1.18)). While studies have identified low-penetrance alleles that associate with breast cancer risk in carriers of *BRCA1* mutations and carriers of *BRCA2* mutations [32],[33], specificities have also been detected [31],[33],[40]. Here, the results of linkage and association studies support a potential, specific genetic interaction between *BRCA1* and *HMMR* (high- and low-penetrance mutations, respectively), which could highlight a BRCA1-RHAMM function altered in familial and sporadic breast carcinogenesis.

Analysis of public gene expression datasets suggests that the rs299290 risk allele is associated with *HMMR* germline over-expression (see also Table S3) [23]. However, while the rs299290 variant represents a missense change predicted to be benign (V368A; concordant predictions for PolyPhen-2 [41] and SIFT [42] were obtained), it is in linkage disequilibrium (according to HapMap Caucasians data: *D′* = 1 and *r^2^* = 0.48) with rs299284 (R92C in Entrez accession number NP_036616), which is predicted to be damaging. The minor allele frequencies of rs299290 and rs299284 in HapMap Caucasian individuals are 29% and 16%, respectively. Since rs299284 is at the fourth base position of *HMMR* exon 5, we evaluated the potential alteration of the splicing pattern of this exon or the ratio of the alternative exon 4. Notably, exon 4 spans the microtubule-binding domain and has been shown to be skipped with progression of myeloma and breast cancer [43],[44]. However, no differences were observed when analyzing the splicing pattern of both exons in lymphocytes from 10 *BRCA1* mutation carriers (Figure S1) and in public transcriptome sequence datasets (unpublished data). Therefore, further work may be warranted to conclusively define the causal mutation(s) and its potential alteration of RHAMM levels or function.

### Association by ER Timor Status and Cytoskeletal Reorganization during Epithelial Apicobasal Polarization

Breast tumors arising in *BRCA1* mutation carriers are typically ER-negative, whereas most tumors in *BRCA2* mutation carriers and sporadic cases are ER-positive [11],[12]. Given the evidence above, we next evaluated whether *HMMR* variation was associated with ER tumor status in *BRCA1* and/or *BRCA2* mutation carriers. In data provided by several CIMBA groups (Text S1), no ER-positive tumors were observed among rare rs299290 homozygotes in *BRCA1* mutation carriers (*p*_interaction_ = 0.006), whereas this bias was not observed in *BRCA2* mutation carriers (*p*_interaction_ = 0.95) (Table S4). That is, despite the expected differences in the frequency of tumor types between the two sets of carriers, heterogeneity was observed in the distribution of rs299290 genotypes in *BRCA1*, but not *BRCA2*, mutation carriers. This result further suggests an interaction between *BRCA1* and *HMMR* that influences or regulates differentiation of breast luminal epithelium. On the basis of these observations and the published data presented above, we next investigated the relationship between *BRCA1*/BRCA1 and *HMMR*/RHAMM regulating apicobasal polarization (hereafter polarity/polarization).

The growth of nonmalignant human mammary epithelial cells, such as MCF10A and HMT3522 S1, within three-dimensional cultures containing reconstituted basement membrane (rBM) recapitulates aspects of the terminal differentiation of mammary luminal epithelia, including apicobasal polarization, growth arrest, and milk production [45],[46]. The cyst-like polarized structures (hereafter termed acini) formed by these cell types may, however, vary in the nature or degree of polarization and tight junction formation and, unlike heterotypic cultures of stromal and epithelial cells [47], do not form bilayered cellular organizations [48]. Importantly, disruption of BRCA1 function through shRNA-mediated depletion impairs differentiation and promotes proliferation of MCF10A cells within rBM [14]. This seminal observation has been supported by evidence from other models for differentiation [15],[16] and the examination of human mammary epithelial cell populations [17]. However, to date, the molecular contributions of BRCA1 to apicobasal polarization are largely unknown. Thus, we utilized the growth of MCF10A cells in rBM as a model for polarization, as determined by the apical localization of centrosomes, basal deposition of CD49f (also known as α6-integrin) and reduced expression of vimentin (VIM), an intermediate filament associated with the basal lineage [49]. These attributes were also captured through quantitation of acini size and circularity or shape factor (Figure S2).

**Figure 2:**
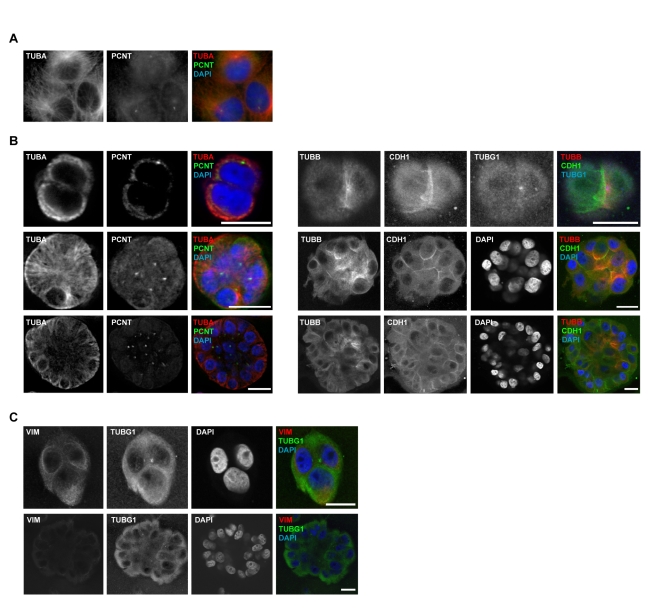
Centrosome microtubule assembly is altered as MCF10A are cultured on two- or three-dimensional systems. (A) Microtubule density (α-tubulin, TUBA) is concentrated around centrosomes (PCNT) within adherent MCF10A. (B) When grown in rBM, microtubule density (TUBA and β-tubulin, TUBB) is initially (top panels, days 1–3 of culture) concentrated around centrosomes (deconvolved *z*-slices from epifluorescence microscopy images, left panels; confocal microscopy images, right panels; E-cadherin, CDH1; and TUBG1). Upon apical localization of centrosomes (middle panels, days 4–7), microtubule density is amplified at cell-to-cell contacts, as determined by CDH1. This organization is maintained through acinar morphogenesis and lumen formation (bottom panels, after day 10). Scale bars represent 20 µm. (C) Reorganization of VIM intermediate filaments during apicobasal polarization in rBM culture. Confocal images were acquired with equivalent settings to allow comparison of intensities. Scale bars represent 20 µm.

As BRCA1 and RHAMM functions may intersect at the organization of microtubules and centrosomes, these structures were first examined in MCF10A cells grown on two-dimensional (i.e., plastic) versus three-dimensional (i.e., rBM) cultures. In plastic, microtubules were assembled at centrally located centrosomes (Figure 2A). During polarization in rBM, however, microtubule organization transitioned from centrosome-dependent assembly in early stages of culture to concentrate at non-centrosome sites, such as regions of cell-to-cell contact, in late stages (Figure 2B). Centrosomes were repositioned from the outside of cell clusters to apical surfaces and the eventual site of the lumen (Figure 2B). This organization was maintained in polarized acini (Figure 2B) and is comparable to the apical position of centrosomes in mammary epithelial cells in vivo (see also Figure S3A) [50]. Thus, polarization of MCF10A is associated with a transition in the organization of microtubules from centrosome to non-centrosome sites, consistent with observations in other epithelial cells or tissues [9],[10].

Complementary to the study of microtubules, the dynamics of VIM were also examined during polarization. In accordance with a shift from basal to luminal cytoskeletal structures, VIM abundance was reduced concurrent with the transition to non-centrosome-dependent microtubule organization (Figure 2C) and the deposition of CD49f (Figure S2). Therefore, polarization requires dynamic cytoskeletal organization. However, the mechanistic contribution of BRCA1 to this process remains unknown.

### Cytoskeletal Reorganization Is Influenced by BRCA1 and Microtubule-Associated Factors within Polarized Epithelia

**Figure 3:**
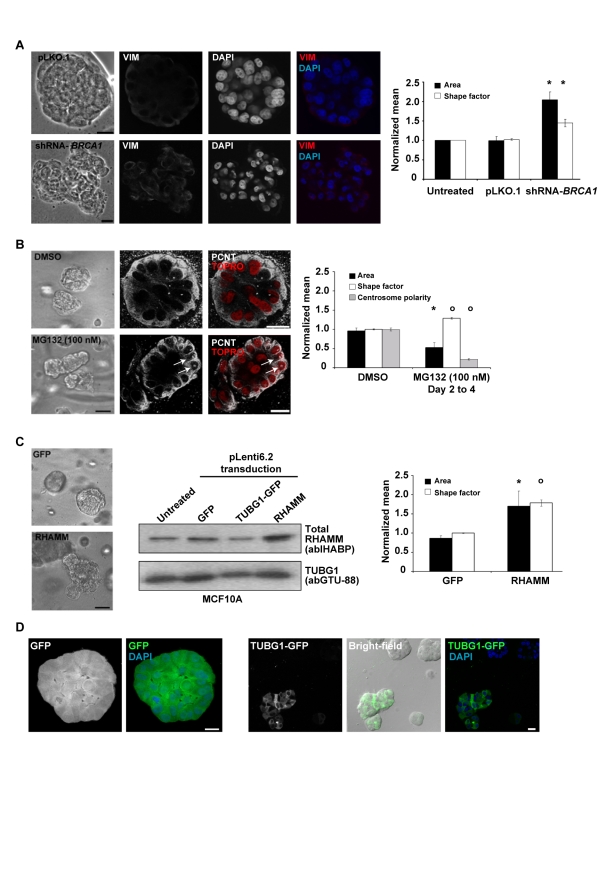
BRCA1 and RHAMM function in epithelial apicobasal polarization. (A) BRCA1 depletion (shRNA-mediated assay) impairs polarization. Representative bright-field images are shown from control vector pLKO.1 and shRNA-*BRCA1* (pLKO.1-based) transduced cultures. Scale bars represent 20 µm. Confocal microscopy images of VIM immunostaining in control and BRCA1-depleted acini are shown. The graph shows results for the area and shape factor measures from four independent experiments. Asterisks indicate significant differences (two-sided *t* test *p*<0.05) from controls. (B) Proteasome inhibition (MG132 100 nM) significantly altered acini area and shape factor, and centrosome structure and polarity. Representative bright-field images are shown from DMSO- or MG132-treated cultures. Confocal microscopy images for centrosome structure and polarity (PCNT) in acini following proteasome inhibition, with nuclei counterstained with TOPRO (false color red), are shown. Arrows indicate altered centrosome structures. The graph shows the results of at least three independent experiments. Average centrosome polarity was determined from PCNT signal position within acini relative to nuclei. Across treatments, 33 acini were analyzed, averaging 24.7 centrosomes and nuclei/acini. Circles indicate significant differences (two-sided *t* test *p*<0.005) to controls. (C) RHAMM over-expression (pLenti6.2-driven) impairs polarization. Representative bright-field images are shown from control GFP vector or RHAMM (pLenti6.2-) transduced cultures. Middle panel, Western blot analysis for RHAMM over-expression. The graph shows the results of four independent experiments. Values were normalized to untreated cultures within experiments and differences evaluated from GFP controls. (D) TUBG1-GFP over-expression (pLenti6.2-driven) impairs polarization. MCF10A were transduced with GFP or TUBG1-GFP expression constructs, selected with blasticidin and fluorescence-activated cell sorting. Sorted cells were then analyzed for polarization in rBM and the resulting acini examined by bright-field and epifluorescence microscopy. GFP over-expression permitted polarization (left panels). However, acini over-expressing TUBG1-GFP were unable to polarize (representative acini at bottom left in the right panels). Blasticidin-resistant clones with low TUBG1-GFP expression formed normal acini with lumen, as indicated by DAPI (top right acini in the bright-field image). Scale bars represent 20 µm.

As BRCA1 down-regulates centrosome microtubules by targeting microtubule-associated factors for proteasome-dependent degradation [20],[21], we hypothesized that this activity may be important for the transition to non-centrosome-dependent assembly that is essential for polarity [9],[10]. To evaluate this hypothesis, we first examined the impact of BRCA1 depletion on polarization and cytoskeletal structures. In agreement with a previous report [14], transduction of lentiviral-based shRNAs against *BRCA1* expression (shRNA-*BRCA1*) impaired polarization; observed acini in this condition were, on average, significantly larger and less circular than controls (Figure 3A). Results were similar following transduction of individual (two different sequences) or pooled shRNAs, with transient or stable shRNA expression assays, and over a time course of one or two weeks (Figures S4 and S5). In addition, VIM and CD49f expression were increased and reduced, respectively, in acini depleted of BRCA1 relative to controls (Figure 3A and S6). Thus, loss of BRCA1 function may impair polarization by altering intracellular cytoskeletal organization, resulting in intermediate filament content consistent with the characteristic basal-like tumor type.

While *BRCA1* haploinsufficiency does not preclude the formation of a functional luminal layer, the cytoskeletal structure within luminal epithelia from *BRCA1* mutation carriers might be compromised. Accordingly, histologically normal breast tissue from *BRCA1* mutation carriers revealed elevation of ALDH1-positive cells with reduced expression of cytoskeletal markers (cytokeratins 18 and 14) and ER [16]. Given these observations, we evaluated TUBG1 staining, as a centrosome marker, in breast tissue paraffin sections from four affected *BRCA1* mutation carriers. Three hyperplastic lesions were identified that showed abnormal localization of the centrosome when considering their respective nuclei and lumen (Figure S3B). Although the number of samples is limited, these results agree with the loss of polarity observed in MCF10A cells after BRCA1 depletion.

Next, we used chemical and biological tools to dissect the mechanistic contribution of BRCA1 to MCF10A polarization. Should polarization require BRCA1-mediated reduction in microtubule assembly at the centrosome, proteasome inhibition may disrupt this transition, even in the presence of BRCA1. When grown in rBM, the major phenotypic response of MCF10A cells to proteasome inhibition (MG132, see Materials and Methods) was growth ablation and/or retardation (unpublished data). However, exposure to 100 nM of MG132 for short periods of time resulted in abnormal acini that deviated from circularity with impaired centrosome apical polarity (Figure 3B). Additionally, proteasome inhibition altered centrosome structures, resulting in diffuse and enlarged pericentrin (PCNT) organization (Figure 3B, arrows). Thus, proteasome inhibition phenocopies aspects of BRCA1 depletion, which suggests that proteolytic degradation of BRCA1-target(s), such as RHAMM [23], may be critical for polarization. To further evaluate this, we examined the influence of BRCA1 depletion and proteasome inhibition on the abundance of RHAMM and aurora kinase A (AURKA), a defined proteasome target [51]. Importantly, both proteasome inhibition and BRCA1 depletion increased the abundance of RHAMM (Figure S7), which is also consistent with observed RHAMM over-expression in breast cancer cell lines derived from *BRCA1* mutation carriers [23]. BRCA1 depletion, however, did not alter AURKA levels (Figure S7). Thus, RHAMM abundance, which is responsive to both BRCA1 depletion and proteasome inhibition, may play a pivotal role in the polarization necessary for differentiation.

One critical role of RHAMM/xrhamm may be the accumulation of TUBG1/tubg1 at the centrosome to influence microtubule assembly [26],[27] and interphase microtubule dynamics [28]. To further determine whether accumulation of microtubule-associated factors was sufficient to disrupt polarization, RHAMM and TUBG1, tagged with the green-fluorescent protein (GFP; TUBG1-GFP), were constitutively over-expressed in MCF10A cultures. Even in the presence of BRCA1, over-expression of RHAMM produced significantly larger and less circular acini (Figure 3C). Accordingly, over-expression of TUBG1-GFP (but not GFP alone) impaired centrosome apical localization and resulted in grape-like cell clusters with aberrant mitotic spindles (Figure 3D). Therefore, increases in microtubule-associated factors–through BRCA1 depletion, proteasome inhibition, or over-expression of centrosome proteins targeted by BRCA1-dependent ubiquitination–impair polarization. If decreased microtubule assembly at centrosomes is fundamental to BRCA1-mediated polarization, concurrent depletion of BRCA1 and associated factors may recover this process.

### Interactions between *AURKA*, *BRCA1*, *HMMR*, and *TPX2* Regulate Polarization

**Figure 4:**
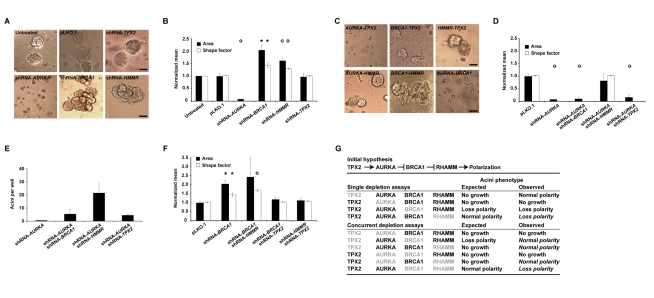
Genetic interactions influencing epithelial apicobasal polarization. (A) shRNA-mediated depletion of centrosome components impairs polarization. Representative bright-field images are shown for results of untreated and control vector pLKO.1, shRNA-*AURKA*, shRNA-*BRCA1*, shRNA-*HMMR*, or shRNA-*TPX2* transduced cultures of MCF10A cells in rBM. Magnification is equivalent for all images and scale bars represent 20 µm. (B) Acini architecture was quantified from bright-field images of cultures treated as described above. For comparison between experiments, all values were normalized to untreated cultures within experiments and differences assessed statistically relative to pLKO.1. Shape factor values for single cells, or small clusters, are not plotted. The graph shows the results of at least four independent experiments. For all graphs, asterisks and circles indicate significant differences (two-sided *t* test *p*<0.05 and *p*<0.005, respectively) from controls (pLKO.1). (C) Representative bright-field images of acini from concurrent depletions (shRNA-mediated) as indicated. (D) *AURKA-HMMR* interact in the regulation of polarization: *HMMR* depletion rescues the abnormality seen in the shRNA-*AURKA* assay. Graph shows the results of three independent experiments. (E) Quantification of acini per well confirms the genetic interaction between *AURKA* and *HMMR*. Graph shows the results of duplicate experiments. (F) *TPX2* depletion is suppressive to abnormalities caused by shRNA-*BRCA1* and shRNA-*HMMR*. Graph shows the results of at least three independent experiments. (G) Prior to the shRNA assays, published data proposed the hypothesis of a signaling pathway from TPX2 to RHAMM regulating polarization; degradation of the microtubule-associated factor RHAMM, through BRCA1, was predicted as key to polarization. However, several observations from the single and concurrent depletion assays (depleted proteins are indicated in grey font) diverged from the expected results (divergent observations are italicized). RHAMM depletion impaired polarization in a manner that was rescued by concurrent depletion of AURKA or TPX2, but not BRCA1. On the other hand, concurrent depletion of BRCA1 and TPX2 revealed normal acini.

Active AURKA phosphorylates BRCA1 to influence interphase microtubule assembly at the centrosome [52]; in turn, AURKA is activated by a complex with targeting protein for *Xenopus* kinesin-like protein 2 (TPX2) [53]. Therefore, to comprehensively examine the molecular determinants of BRCA1-mediated polarization, we evaluated the consequences of single and concurrent depletions of AURKA, BRCA1, RHAMM, and TPX2 expression. As with experiments targeting BRCA1 expression, depletion of AURKA, RHAMM, and TPX2 was performed using individual and pooled shRNAs, with transient or stable shRNA expression assays, and over a time course of one or two weeks (Figures S4 and S5). Note that depletions were not complete for any target, so results should be interpreted in the context of partial loss-of-function. Depletion of TPX2 did not impair growth, did not disrupt polarization, and only slightly reduced the average acini area (Figures 4A,B, S4, and S5). However, depletion of AURKA significantly reduced two- and three-dimensional cellular growth (Figures 4A,B, S4, and S5), which parallels the effect of a small molecule inhibitor [54]. Finally, depletion of RHAMM induced visible scattering in two-dimensional growth (Figure S4B) and increased the area and altered the circularity of acini (Figures 4A,B, S4, and S5). These results were further supported by observations of VIM and CD49f immunostaining in acini (Figure S6). Thus, alteration of RHAMM levels by over-expression or depletion impairs polarization in a similar manner to BRCA1 depletion, which suggests critical regulation of RHAMM in this process.

Having established the effects of single depletions, we investigated the genetic interactions that regulate polarization. Using concurrent, transient assays with pooled shRNAs, we identified interactions between *AURKA* and *HMMR* (type double nonmonotonic [55]), *BRCA1* and *TPX2* (type suppressive [55]), and *HMMR* and *TPX2* (type suppressive [55]) that regulate polarization (Figure 4C). Notably, simultaneous depletion of BRCA1 and RHAMM did not rescue the polarity defects of the corresponding single depletion assays (Figure 4C, 4F, and 4G). In fact, equivalent acini alterations were observed. As down-regulation of a microtubule-associated factor (i.e., RHAMM) did not recover BRCA1 depletion, a more complex regulation of cytoskeletal reorganization during polarization may exist.

In contrast to single depletions, simultaneous reduction of AURKA and RHAMM levels recovered normal acini formation (Figure 4C–G), possibly implying a negative regulatory relationship between RHAMM abundance and AURKA activity. Although mechanistic insight into this relationship is lacking, RHAMM depletion also protects against small-molecule inhibition of AURKA in a different cell model [56]. Notably, depletion of TPX2, the major activator of AURKA [53], recovered normal acini formation with concurrent depletion of either BRCA1 or RHAMM (Figure 4C, 4F, and 4G). Together, these genetic interactions suggest that a balance between AURKA-TPX2 and BRCA1-BARD1 activities, mediated by RHAMM, may determine proliferation and polarization.

Should AURKA antagonize BRCA1-BARD1 ubiquitination activity to promote centrosome-dependent microtubule assembly [52], AURKA depletion may amplify the degradation of BRCA1-targeted molecules. As presented above, we confirmed this relationship by examining RHAMM abundance, which was augmented by BRCA1 depletion (Figure S7B and S7C). Consistently, AURKA depletion reduced RHAMM levels (Figure S7C), while simultaneous depletion of AURKA and BRCA1 recovered RHAMM to control levels (Figure S7C). Taken together, these data indicate a critical relationship between AURKA and BRCA1 in regulating RHAMM abundance and, thus, polarization.

### pT703-RHAMM Negatively Regulates AURKA Activity through Nuclear Sequestration of TPX2

**Figure 5:**
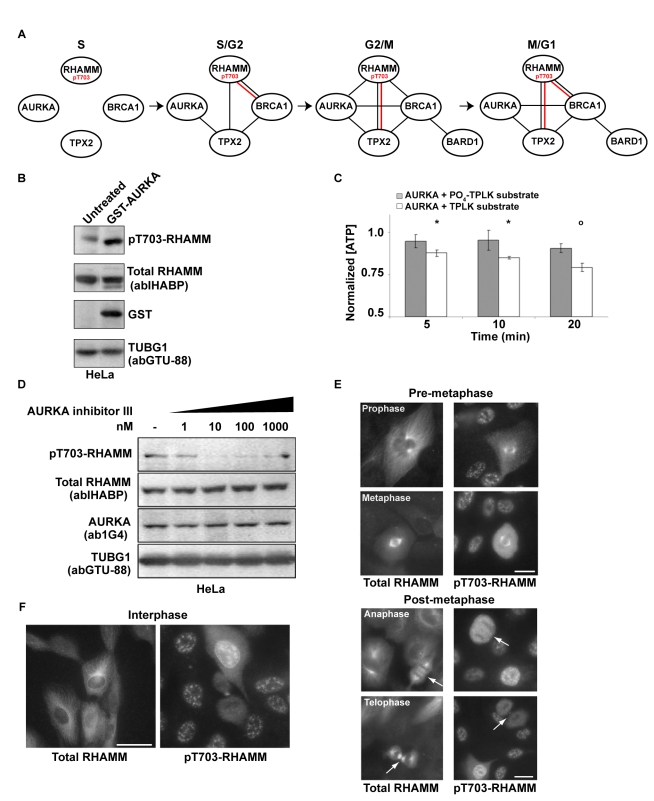
pT703-RHAMM functionally connects AURKA with BRCA1 and TPX2. (A) Molecular diagram of co-immunoprecipitation results (Figure S8) between centrosome module components across the cell cycle, including complexes from pT703-RHAMM IPs (shown in red). (B) Over-expression of GST-AURKA increases pT703-RHAMM. Lysates from HeLa cells, untreated or transfected with GST-AURKA, were immunoblotted for the indicated proteins (GST-AURKA detected by anti-GST). (C) Position T703 of RHAMM is an AURKA substrate in vitro. When normalized to reactions lacking substrate, the combination of recombinant AURKA, ATP, and a T703-containing peptide substrate (acetyl-CKENFALK(T)PLKEGNT-amide) resulted in time-dependent consumption of ATP as measured by luminescence. In contrast, a pre-phosphorylated (PO_4_) T703-containing peptide (acetyl-CKENFALK(PO_4_-T)PLKEGNT-amide) showed muted AURKA activity. Asterisk and circles indicate significant differences (two-sided *t* test *p*≤0.05 and *p*<0.005, respectively) relative to control condition (no peptide). (D) AURKA inhibition results in specific loss of pT703-RHAMM. Lysates of HeLa treated with graded concentrations of an AURKA inhibitor (see Materials and Methods) were immunoblotted for the indicated endogenous proteins. (E) pT703-RHAMM cellular immunoreactivity is lost post-metaphase. Consistent with previous reports [23],[63],[76], total RHAMM decorates all microtubule structures throughout mitosis. In contrast, pT703-RHAMM is lost, or reduced, on microtubule structures after metaphase (arrows). Interphase cells within the field of view indicate specific loss of pT703-RHAMM post-metaphase. The indicated mitotic stage was determined by microtubule organization and DNA condensation (unpublished data). (F) pT703-RHAMM localizes to nuclear compartments. pT703-RHAMM localizes to the nucleus and nuclear envelope. An in-frame post-metaphase cell indicates that nuclear labeling is specific to interphase. Magnification is equivalent for all images and scale bar represents 10 µm.

Complementary analyses suggest a *BRCA1*-*HMMR* interaction linked to early-onset, ER-negative breast tumorigenesis, while polarization studies suggest that RHAMM abundance is central to BRCA1 and AURKA activities. As AURKA function relies upon a physical association with TPX2 [53], we next investigated protein complexes through the cell cycle to determine the relationship between RHAMM abundance and AURKA activity. Consistent with prior reports [26],[27], co-immunoprecipitation assays confirmed strong reciprocal interactions between RHAMM and TPX2 during periods of microtubule re-organization (G2/M, spindle assembly, and M/G1, spindle disassembly) (Figures 5A and S8). Importantly, immunoprecipitation of BRCA1-associated or TPX2-associated protein complexes revealed mobility-shifted RHAMM species suggestive of phosphorylation (Figure S8).

Threonine 703 (T703) is an evolutionarily conserved phosphorylated residue in RHAMM [57] similar to a consensus aurora kinase Ipl1p site [58]. We carried out complementary analyses to test this site as an AURKA substrate. Ectopic expression of GST-AURKA increased levels of phosphoT703-RHAMM (pT703-RHAMM) (Figure 5B), as detected by a novel polyclonal antibody (Figure S9 and Materials and Methods). In MCF10A cells, AURKA abundance and activity determined total RHAMM as well as pT703-RHAMM levels (Figure S10A). An in vitro kinase assay with recombinant AURKA confirmed T703-RHAMM site-specific activity (Figure 5C). Finally, pT703-RHAMM was reduced in a dose-dependent manner with AURKA inhibition (Figures 5D and S10B) and with mitotic progression (Figure 5E), which is consistent with AURKA degradation in anaphase [51].

**Figure 6:**
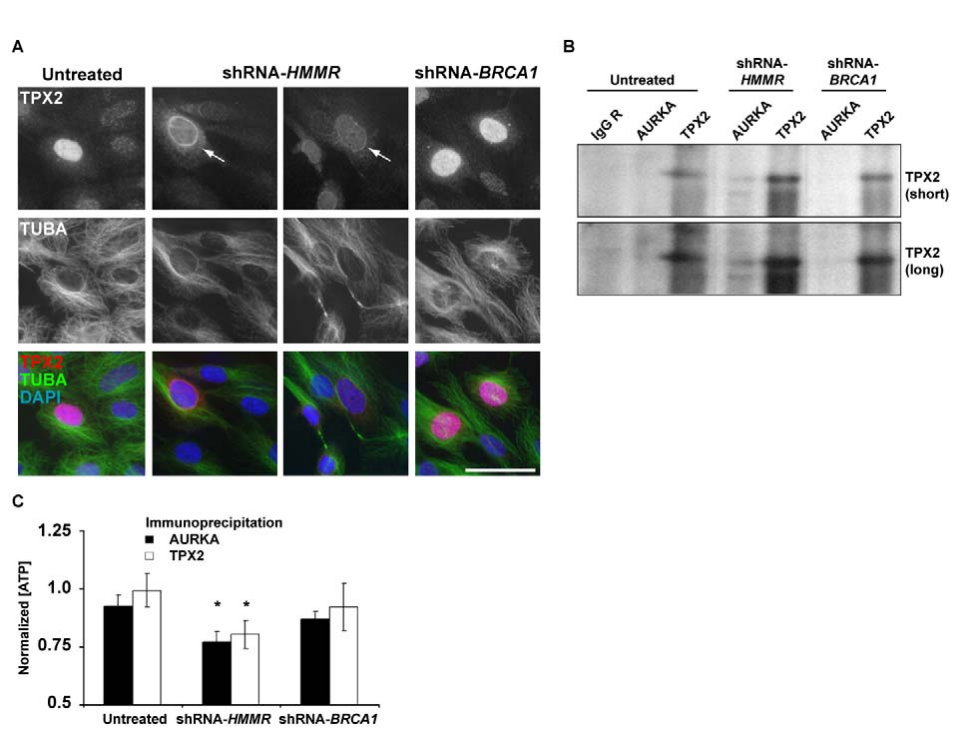
RHAMM depletion alters TPX2 localization and AURKA activity. (A) Depletion of RHAMM, but not BRCA1, results in re-localization of TPX2 from the nucleus to the nuclear envelope and cytoplasm (arrows). With RHAMM depletion, microtubule organization is less focused and radial. Scale bar represents 20 µm. (B) RHAMM depletion alters AURKA-TPX2 association. In triplicate experiments, MCF10A were untreated or depleted of BRCA1 or RHAMM, and lysates were immunoprecipitated with AURKA, TPX2, or control IgG antibodies. Compared to untreated or BRCA1-depleted samples, RHAMM depletion resulted in an increase of TPX2 co-precipitated with AURKA. Short and long Western blot exposures are shown. (C) RHAMM depletion alters AURKA activity. Immunoprecipitation beads from triplicate experiments were analyzed for kinase activity using luminescent detection of ATP. Luminescence values were normalized to those obtained for beads precipitated with control IgG. Beads from untreated lysates precipitated with AURKA but not TPX2 antibodies demonstrated modest kinase activity. Depletion of RHAMM led to a significant increase in kinase activity with both AURKA and TPX2 precipitation (asterisks indicate one-sided *t* test *p*<0.05). Graph shows means and standard errors from triplicate experiments.

Importantly, while total RHAMM was predominantly cytoplasmic with enrichment at microtubules and centrosomes, pT703-RHAMM localized to interphase nuclei (Figure 5F). This observation prompted the hypothesis that pT703-RHAMM maintains homeostasis of AURKA activity by sequestering TPX2 in the nucleus. Consistent with this hypothesis, pT703-RHAMM immunoprecipitated with TPX2 during periods of high AURKA activity (G2/M as previously described [52]) (Figures 5A and S8), while RHAMM depletion not only redistributed TPX2 to the cytoplasm and nuclear envelope (Figure 6A) but also increased the level of TPX2 immunoprecipitated with AURKA (Figure 6B). In addition, RHAMM depletion increased AURKA activity as measured by an in vitro kinase assay with beads from AURKA and TPX2 immunoprecipitations (Figure 6C). Collectively, these data indicate that RHAMM maintains AURKA homeostasis as a kinase substrate that, when phosphorylated, negatively regulates AURKA-TPX2 complex formation. Moreover, these results illustrate how depletion of RHAMM alone, or in combination with BRCA1, impairs polarization through augmentation of AURKA activity.

### pT703-RHAMM Expression in *BRCA1* Mutant Breast Cancer Cells and Tumors; A Mechanistic Model for Polarization and Increased Risk of Breast Cancer

**Figure 7:**
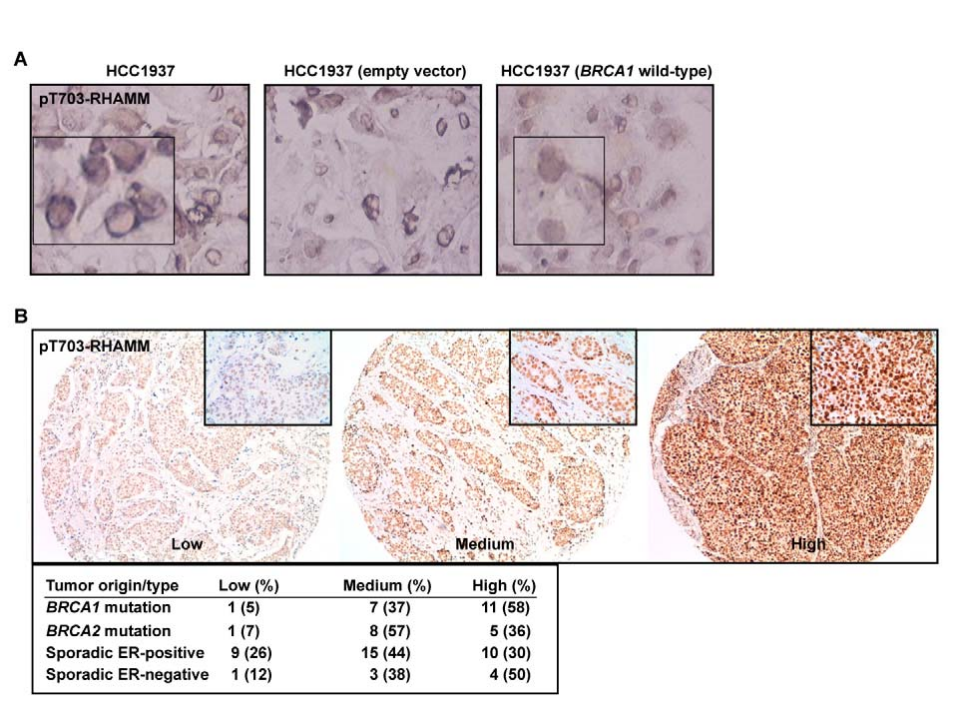
pT703-RHAMM expression in *BRCA1* mutant breast cancer cells and tumors. (A) pT703-RHAMM staining is strong at the nuclear envelope of HCC1937 cells (*BRCA1* mutated or transduced with an empty vector; left and middle panels, respectively) but homogeneous nuclear in *BRCA1* wild-type reconstituted cells (right panel). (B) Results of pT703-RHAMM staining scores in primary breast tumors with different *BRCA1/2* mutation and ER status. Results correspond to scores from two pathologists (see Materials and Methods).

The data above indicate that a balance between BRCA1-mediated turnover and AURKA-mediated phosphorylation of RHAMM regulates polarization versus proliferation. To evaluate the link with carcinogenesis, pT703-RHAMM immunochemistry was performed in *BRCA1* mutant breast cancer cells, HCC1937 line, their wild-type reconstituted counterparts, and in primary breast tumors. As a result, pT703-RHAMM staining was revealed to be strong at the nuclear envelope of HCC1937 cells but homogenous and less intense in the nucleus of the reconstituted cells (Figure 7A). Subsequently, high expression of pT703-RHAMM was scored in 58% (*n* = 11) and 50% (*n* = 4) of *BRCA1* mutation carriers and sporadic ER-negative tumors, respectively, but in 36% (*n* = 5) and 30% (*n* = 10) of *BRCA2* mutation carriers and sporadic ER-positive tumors, respectively (Figure 7B). Although this dataset is limited, the results support the indication of an interplay between BRCA1 and RHAMM, which is altered in breast carcinogenesis.

**Figure 8:**
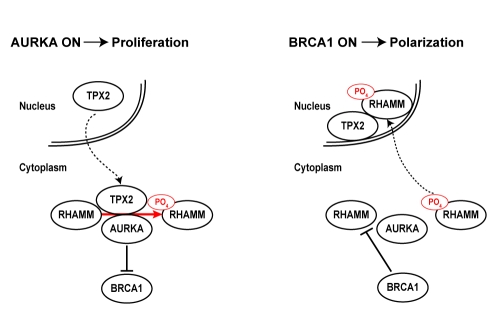
Mechanistic model of interplay between AURKA, BRCA1, RHAMM, and TPX2 that regulates proliferation versus polarization. Proliferation is proposed to be linked to an active (“on”) status of AURKA while differentiation would be linked to an active BRCA1 status, both centered on tight regulation of RHAMM level and localization.

Our data delineate a model in which different types of relationships between high- and low-penetrance breast cancer susceptibility genes and their products regulate the polarization necessary for terminal differentiation of luminal epithelia. That is, BRCA1 and AURKA activities, as regulated by RHAMM and TPX2, control this transition and regulate cellular proliferation and differentiation (Figure 8). In this model, concurrent depletion of BRCA1 and RHAMM does not recover normal acinar morphogenesis because target degradation of RHAMM may be restricted to late phases of polarization. This model is consistent with reduced expression of *AURKA*, *TPX2*, and *HMMR*, but to a lesser extent *BRCA1*, with polarization and growth arrest of nonmalignant mammary epithelial cells, as measured by gene expression profiling (Figure S11A) [59]. Deviation from this pathway, through loss of BRCA1 function or augmentation of microtubule-associated factors, may impair terminal differentiation of luminal epithelia and promote tumorigenesis. Consistently, *HMMR* over-expression might be detectable as early as the transition from normal breast tissue to hyperplasia (Figure S11B) [60]. In our cellular assays for polarization, however, concurrent BRCA1 depletion and RHAMM over-expression did not result in an additive disruption of polarity, perhaps due to the non-additive alteration of RHAMM abundance and variable BRCA1 depletion (Figure S12). According to the model and as stated above, analysis of public gene expression datasets suggests that the rs299290 risk allele is associated with *HMMR* germline over-expression (Table S3) [23]. As the potential splicing alteration by rs299284 might be tissue specific and RHAMM-R92 was used in the over-expression assays, further work may be warranted to define the causal mutation(s) and the alteration of RHAMM function and/or expression level according to the depicted model.

## Discussion

We have investigated gene and protein interactions in a centrosome-cantered module, including *BRCA1*/BRCA1 and *HMMR*/RHAMM, across biological systems ranging from breast cancer risk estimates to cellular phenotypes and cytoskeletal structures. Consistent findings between these systems provide insights into diverse processes and conditions. First, the key role of this module in epithelial apicobasal polarization suggests that genetic variation in its components might influence risk of breast cancer. Accordingly, a common candidate breast cancer-predisposition allele in *HMMR*, originally identified in an Ashkenazi Jewish study [23], may specifically modify breast cancer risk among *BRCA1* mutation carriers. Population-discordant results for *HMMR* [61], and possibly for other components of this module (i.e., *AURKA* [62]), might be due to genetic differences between populations. A recent report has suggested that common genetic variation in genes encoding for centrosome pathway components (excluding *AURKA* and *HMMR*) may frequently influence risk of breast cancer and, notably, includes variants in *TACC3*–a proposed *HMMR* homolog [63]–*TUBG1*, and *TPX2* loci [64]. Our results highlight the importance of conducting comprehensive evaluations of the interactions between cancer susceptibility genes and their products across systems to delineate the potential relationship with carcinogenesis [65].

A unifying mechanism of breast carcinogenesis linked to BRCA1 loss-of-function should provide a comprehensive explanation for the observed accumulation of stem and luminal progenitor cells [17]–[19], and for the characteristic pathological features of the corresponding tumors [11],[12]. The results of our study suggest that BRCA1 promotes the polarization necessary for luminal differentiation, in part, by orchestrating the dynamic transition to microtubule assembly at non-centrosome sites (i.e., cell-to-cell contacts). Transition to microtubule anchorage at adherens junctions regulates epithelial cell-to-cell contacts [9] that, in turn, instruct mammary stem cell fate and differentiation [66]. Thus, the switch to non-centrosome-dependent assembly of microtubules may be essential in discriminating between proliferating and differentiated cells, as also observed recently in myoblasts [67] and neurons [68]. Should BRCA1 function(s) promote this transition in mammary stem/progenitor cells, impaired luminal differentiation in *BRCA1* mutation carriers and a propensity to develop basal-like tumors with elevated proliferative capacity would be expected. Further studies using different polarization and/or differentiation cellular models may be warranted to corroborate the depicted mechanism.

Proliferation is promoted by activated AURKA but, as occurs in tightly synchronized cell cycle events [69], possibly regulated through a negative feedback loop as identified in this study. In preparation for mitotic spindle assembly, AURKA promotes centrosome-dependent microtubule assembly by suppressing BRCA1-dependent ubiquitination [52], which involves BRCA1 phosphorylation at S308 [70]. Subsequently, terminal differentiation is proposed to be mediated by BRCA1 activation and RHAMM degradation. Accordingly, BRCA1 depletion increases the clonogenic potential of mammary epithelia [14]–[16], while a BRCA1 S308A mutant alters embryonic stem cell differentiation [71]. Moreover, RHAMM abundance may be central to the balance between AURKA-TPX2 and BRCA1-BARD1 activities during polarization; consistently, depletion of RHAMM also impairs ciliary differentiation of human respiratory epithelial cells [72]. However, key components of the depicted molecular wiring diagram are probably missing, such as a phosphatase that regulates AURKA-mediated modification of BRCA1 and RHAMM. Additionally, loss of BRCA1 function is likely to alter complementary pathways such as the regulation of epithelial-mesenchymal transition [19] and androgen receptor signaling [73].

A key question remains regarding the significance of BRCA1 function to stem/progenitor differentiation and *BRCA1* haploinsufficiency. Examination of histologically normal breast tissue in *BRCA1* mutation carriers revealed cellular foci expressing stem cell markers and lacking cytoskeletal structures characteristic of luminal epithelia [16]. Here, we suggest an alteration of polarization in preneoplastic lesions. Neither of these presentations is as severe as those observed in murine mammary epithelial tissues reconstituted from human cells depleted of BRCA1 [19]. Thus, *BRCA1* dose or mutation type may distinctly affect the tissue architecture and function, leading to differences in the accumulation of stem or progenitor cells, and the resulting tumor type [74]. More detailed examination of mammary gland histology and function may reveal specificities in *BRCA1* mutation carriers reflective of a gradient in the disruption of luminal differentiation. To summarize, this study describes a mechanistic model in which high- and low-penetrance breast cancer susceptibility genes and their products are connected through a series of genetic, molecular, and functional interactions that, when perturbed, alter proper epithelial apicobasal polarization and may lead to an increased risk of breast cancer.

## Materials and Methods

### Ethics Statement

*BRCA1* and *BRCA2* mutation carriers were recruited under the CIMBA initiative following approval of the corresponding protocols by institutional review boards or ethics committees at each participating centre, as described [32],[33].

### Study Samples, Genotyping, and Statistical Analyses

Study acronyms are detailed in Table S2. The NICCC centre in Israel followed similar protocols and similar approval processes. Deviation from Hardy-Weinberg equilibrium was evaluated among unrelated participants separately for each study. Risk estimates and significance testing were computed using standard and weighted Cox regression models [35] that included centre, country, and birth cohort (<1940, 1940–1949, 1950–1959, and ≥1960) as stratification factors and ethnicity as the covariate for adjustment. A robust variance estimate was used to account for familial correlation. Time to diagnosis of breast cancer from birth was modeled by censoring at the first of the following events: bilateral prophylactic mastectomy, breast cancer diagnosis, ovarian cancer diagnosis, death, and last date known to be alive. Participants were considered affected if they were censored at breast cancer diagnosis and unaffected otherwise. The weighted cohort approach involves assigning weights separately to affected and unaffected individuals such that the weighted observed incidences in the sample agree with established estimates for mutation carriers [35]. This approach has been shown to adjust for the bias in the HR estimates resulting from the ascertainment criteria used, which leads to an over-sampling of affected women. Weights were assigned separately for carriers of mutations in *BRCA1* and *BRCA2* and by age interval (<25, 25–29, 30–34, 35–39, 40–44, 45–49, 50–54, 55–59, 60–64, 65–69, ≥70). Polymorphism data were analyzed as a three-group categorical variable (codominant model) and using restricted inheritance models (log-additive, dominant and recessive). The *p* values were derived from the robust score test. All statistical analyses were carried out using R software. Linkage analysis was performed with GENEHUNTER version 2.1 [75].

### Cell Culture

HeLa (American type culture collection, ATCC), 293FT (Invitrogen), and MCF10A (ATCC) were cultured in media as recommended. For growth factor-reduced experiments, media (HuMEC from Invitrogen or HMEC from Lonza) contained 1/3 recommended hEGF. Growth in rBM (Cultrex from Trevigen or Geltrex from Invitrogen) followed embedded or on-top techniques as described [45]. MCF10A were embedded in rBM for proteasome inhibition (MG132; Sigma-Aldrich) experiments; for other endpoints, embedded and on-top conditions were equivalent. For rBM growth, MG132 or equivalent DMSO volumes were added to media at seeding, or as indicated, for two days. For proteolysis protection, MG132 (1.5 µM) was added for 3 h prior to lysis. For AURKA inhibition, a commercially available AURKA inhibitor (C1368; Sigma-Aldrich) was titrated and used at 100 nM. For TUBG1-GFP expression, MCF10A were transduced with a lentiviral-based vector, sorted, and selected for blasticidin resistance (pLenti6.2/EmGFP-DEST, Invitrogen).

### Constructs, Transductions, and Transfections

For expression in MCF10A, RHAMM and TUBG1-GFP were subcloned into pDONR223 (Invitrogen), sequenced, and transferred to pLenti6.2/V5-DEST following the manufacturer's instructions (Invitrogen). All constructs maintained native stop codons. Depletion assays used MISSION shRNA sequences (Sigma-Aldrich), shown in Table S5. The lentiviral packaging, envelope, control, and GFP expression plasmids (psPAX2, pMD2.G, non-hairpin-pLKO.1, scrambled-pLKO.1, and pWPT-GFP) were purchased from Addgene. Production and collection of lentiviral particles followed a modified Addgene protocol. Initial viral titres >5×10^5^/ml were confirmed by Lenti-X GoStix (Clontech) and supernatants were then concentrated by ultracentrifugation or Lenti-X Concentrator (Clontech) and stored at −80°C. Concentrated viral supernatants were titrated for optimal inhibition of target gene products, by immunoblot at 5 d, and MCF10A survival. For shRNA-mediated depletion of BRCA1, four shRNA species were purchased and tested; these sequences were distinct from that previously described [14]. For depletion of AURKA, RHAMM, and TPX2, five shRNA constructs were purchased for each gene (Sigma-Aldrich). Initial experiments used combinations of shRNAs targeting individual genes (up to five sequences per gene) at a multiplicity of infection of five. For confirmation experiments, individual and redundant constructs were identified with high knockdown efficacy. Two shRNA sequences effectively reduced the expression of AURKA (5′-ACGAGAATTGTGCTACTTATA-3′ and 5′-CCTGTCTTACTGTCATTCGAA-3′), BRCA1 (5′-CACCTAATTGTACTGAAT-3′ and 5′-TACAAGAAAGTACGAGAT-3′), and RHAMM (5′-CGTCTCCTCTATGAAGAACTA-3′ and 5′-GCCAACTCAAATCGGAAGTAT-3′), respectively. These shRNAs have also been independently validated for reduction in mRNA levels by the manufacturer (67%–87% reduction, Sigma-Aldrich). Only one sequence efficiently reduced expression of TPX2 (5′-CCGAGCCTATTGGCTTTGATT-3′). Transient transfection of GST-AURKA in HeLa and MCF10A followed the manufacturers' suggested protocols for Lipofectamine 2000 (Invitrogen) or FuGENE (Roche).

### Biochemical Assays

Synchronization and immunoprecipitation, and immunofluorescence of cells and acini, were performed as described previously [27],[45]. For immunofluorescence analysis, cells were mounted in 90% glycerol/PBS and counterstained with DAPI or TOPRO. The in vitro kinase assay with recombinant HIS-AURKA (PTP055, Cell Science) followed the protocol for the PKLight HTS Protein Kinase Assay Kit (Lonza), as suggested by the manufacturer. Reactions were performed in triplicate. Luminescence values were normalized to the mean value for no-substrate (HIS-AURKA alone) reactions. The activity of endogenous AURKA was determined by performing the kinase assay with ATP, substrate, and immunoprecipitation beads―IgG (negative control), anti-AURKA (positive control), or anti-TPX2 from MCF10A lysates but without recombinant AURKA. Consumption of ATP was determined after incubation for 30 min.

### Antibodies

For total RHAMM, a previously developed and characterized polyclonal antibody (originally named anti-IHABP) was used [24],[76]. The specificity of this antibody has been further evaluated elsewhere (see also Figure S9) [23],[27]. The phosphorylation-specific polyclonal antibody against pT703-RHAMM is a custom reagent generated by New England Peptide. For this antibody, unpurified and purified sera were tested for specificity relative to the RHAMM-total antibody defined above. These assays included immunoblots with shRNA-mediated depletion of RHAMM (Figure S9). Other antibodies included anti-ACTB (A5060, Sigma-Aldrich), anti-AURKA (1G4, Cell Signaling Technology), anti-BRCA1 (SD118, Calbiochem), anti-CD49f (4F10, Millipore), anti-CDH1-Alexa 488 (24E10, Cell Signaling Technology), anti-GST (GE healthcare), anti-MYC (9E10, Sigma-Aldrich), anti-PCNT (Covance), anti-TUBA (B512, Sigma-Aldrich), anti-TUBB-Alexa 647 (9F3, Cell Signaling Technology), anti-TUBG1 (GTU88, Sigma-Aldrich), and anti-VIM (V9, Sigma-Aldrich; or R28, Cell Signaling Technology). Secondary antibodies for immunofluorescence (Alexa) were obtained from Molecular Probes (Invitrogen) and GE Healthcare for immunoblot analysis (HRP-conjugated).

### Image Acquisition and Quantitation

Blind deconvolution with AutoQuant (AutoQuant Imaging Inc.) was performed on images from an Axiovert microscope with Plan-Apochromat 63× objective (Zeiss) (numerical aperture (NA) 1.25) with Z-steps from 0.5–1.0 µm. Alternatively, a Leica DMI 6000 laser scanning confocal microscope equipped with a Leitz HCX Pl-Apo CS 40× oil objective (1.25 NA) captured images as indicated. Epifluorescence images were acquired with an Olympus BX-60 using a Spot camera and Spot3.2.4 software (Diagnostic Instruments). For quantitation of growth in rBM, bright-field images of acini were analyzed for size and shape with ImageJ software (National Institutes of Health). For shape analysis, the square of the inverse of circularity was plotted. The position of the centrosome relative to the lumen, or centre of the cellular cluster, was measured using pericentrin immunofluorescence or TUBG1-GFP in image stacks.

### Breast Cancer Cell Line, Tumors, and Immunochemistry

In order to reconstitute HCC1937 cells with wild-type *BRCA1*, the corresponding full-length open-reading frame was cloned into a retroviral vector S11N and transduced. Assays with the empty vector were used as controls. Cells were fixed in 2% paraformaldehyde and immunochemistry carried out following a standard labelled streptavidin biotin (LSAB) method. For tumors, immunohistochemical staining was performed by the Envision method (Dako, Glostrup, Denmark), with a heat-induced antigen retrieval step. Sections from the tissue array were immersed in 10 mM boiling sodium citrate at pH 6.5 for 2 min in a pressure cooker, and antibodies were used at dilution of 1∶1,500 and 1∶1,000 for pT703-RHAMM and TUBG1, respectively. Scoring for pT703-RHAMM was performed in a blind and independent manner by two pathologists with an initial correlation value of 0.75. Discordant results were then assessed jointly but blind from the genetic status of the samples. Hyperplastic lesions in *BRCA1* mutation carriers were also assessed by both pathologists.

## Supporting Information

Figure S1 Evaluation of potential alteration of the *HMMR* splicing pattern by rs299284 variation. Lymphocytes from 10 *BRCA1* mutation carriers were isolated and DNA and RNA samples purified for genotyping and expression analyses, respectively. Five major homozygotes and five heterozygotes for rs299284 were identified, which revealed complete linkage disequilibrium with rs299290 (unpublished data). Next, reverse transcriptase polymerase chain reactions (30 cycles) were carried out with forward (5′-GACAAAGATACTACCTTGCCTGCT-3′) and reverse (5′-CAGCATTTAGCCTTGCTTCCATC-3′) primers. Sequences were obtained using the reverse primer. Variation at rs29984 (marked by an arrow) does not alter the exon 5 acceptor donor site or the exon 4 inclusion/exclusion ratio. Sample identifiers are shown.
(TIF)

Figure S2 MCF10A cells grown in rBM establish apicobasal polarity and attenuate expression of VIM. (A) Apicobasal polarity and luminal characteristics were confirmed with immunofluorescence. The α6-integrin (CD49f) is deposited at the basal surface upon polarization and the expression of VIM is lost with this transition (acini #1–4). Some acini (#5) fail to polarize and do not deposit CD49f. These acini express VIM and grow larger with diminished circularity. Scale bars represent 20 µm. (B) Acini imaged with bright-field microscopy at low magnification (10×). Scale bar represents 100 µm. Quantitation of acini size and shape with ImageJ software distinguishes polarized from non-polarized acini (quantitation performed on images from 20× magnification are not shown). Normalization to controls allows comparison across replicate experiments. For display purposes, the shape factor was plotted such that values >1 indicate abnormal polarization.
(TIF)

Figure S3 Loss of centrosome polarity in breast hyperplastic lesions of *BRCA1* mutation carriers. (A) Normal luminal structure showing apical localization of the centrosomes (TUBG1) in a tissue donor (unaffected, left panel) and in a *BRCA1* mutation carrier (right panel). Arrows mark properly, lumen-oriented centrosomes in many cells. (B) Three hyperplastic lesions showing loss of polarity in *BRCA1* mutation carriers. Left panels show hematoxylin-eosin staining and the insets correspond to the middle and right panels with results for TUBG1 staining. Loss of polarity is evidenced by the identification of centrosome signals that are not oriented towards the lumen and/or that are located on top of the nuclei (arrows).
(TIF)

Figure S4 Stable depletion of centrosome components determines epithelial apicobasal polarization at an early time-point. (A) Transduction of single or pooled shRNAs targeting the expression of indicated proteins was identified resulting in detectable depletions. Sequences for the indicated shRNA are given in Table S5. (B) Representative bright-field images (low magnification, 20×) for adherent (day 6, plastic) and rBM (day 7, on-top) growth of untreated and puromycin-resistant MCF10A cells transduced with pLKO.1-nonhairpin, shRNA-*AURKA*, shRNA*-BRCA1*, shRNA*-HMMR*, or shRNA*-TPX2*. Scale bars represent 100 µm. (C) Acini architecture was quantified from bright-field images of cultures treated as indicated at 1 week post-plating. For comparison between experiments, all values were normalized to pLKO.1-transduced cultures within experiments. Shape factor ((1/circularity)^2^) values for single cells or small clusters are not plotted (shRNA-*AURKA*). Asterisks and circles indicate significant differences (two-sided *t* test *p*<0.05 and *p*<0.005, respectively) from controls (pLKO.1).
(TIF)

Figure S5 Transient depletion of centrosome components determines epithelial apicobasal polarization at early and late time-points. (A) Representative bright-field images (low magnification, 5×) for rBM (second week, embedded) growth of MCF10A transduced with virus encoding pLKO.1-nonhairpin, or individual (as indicated) or pooled shRNAs targeting the expression of AURKA, BRCA1, RHAMM, or TPX2. Images are scaled equivalently, with the scale bar representing 200 µm. (B) Acini architecture was quantified from bright-field images (10× magnification) of cultures treated as indicated at 1 week post-plating. For comparison between experiments, all values were normalized to pLKO.1-transduced cultures within experiments. Shape factor values for single cells or small clusters are not plotted (shRNA-*AURKA*). Asterisks and circles indicate significant differences (two-sided *t* test *p*<0.05 and *p*<0.005, respectively) from controls (pLKO.1). (C) Acini architecture was quantified from bright-field images (10× magnification) of cultures treated as indicated at 2 weeks post-plating.
(TIF)

Figure S6 Depletion of centrosome components alters the expression profile of VIM and CD49f. In normal acini (control and shRNA-*TPX2*), centrosomes are apically positioned (TUBG1), CD49f is deposited at the basal surface, and VIM is lost. This pattern is altered with depletion of BRCA1 or RHAMM. Acini size and shape are reflective of polarization. Results are shown for the nonhairpin pLKO.1 control, shRNA-*AURKA* (#3), shRNA-*BRCA1* (#34), shRNA-*HMMR* (#4), and shRNA-*TPX2* (pooled) assays.
(TIF)

Figure S7 AURKA and BRCA1 activity determine RHAMM abundance. (A) AURKA and RHAMM are protected by proteasome inhibition. Lysates of MCF10A cells exposed for 3 h to DMSO or MG132 were immunoblotted for the indicated proteins. (B) BRCA1 depletion protects RHAMM, but not AURKA, from degradation. Lysates of MCF10A cells growing in growth factor (GF)-reduced media exposed to MG132 or depleted of BRCA1 were immunoblotted for the indicated proteins. (C) Interplay between AURKA and BRCA1 activity regulates RHAMM abundance. Lysates from MCF10A cultures transduced with control vector pLKO.1, shRNA*-AURKA*, shRNA*-BRCA1*, shRNA*-HMMR*, or shRNA-*TPX2*, or simultaneously with shRNAs-*AURKA/BRCA1*, were immunoblotted for the indicated proteins. shRNA*-AURKA* depletion reduces AURKA as well as RHAMM and TPX2, both cell-cycle-regulated proteins (Figure S8). shRNA-*BRCA1* reduces BRCA1 and specifically increases RHAMM, which is consistent with previous data [23] and a putative role of BRCA1 in proteasome-mediated degradation of RHAMM. shRNA-*HMMR* and -*TPX2* reduce RHAMM and TPX2 levels, respectively. Compared to single depletions, simultaneous depletion of AURKA and BRCA1 normalizes RHAMM levels but not those of TPX2.
(TIF)

Figure S8 Protein complexes during the cell cycle. (A) HeLa cells were harvested at confluence (unsynchronized, Us) or following synchronization with double thymidine (S), double thymidine/nocodazole (G2/M), or release from these blocks for 3 and 5 h (S/G2 and M/G1, respectively). Synchronization was confirmed by bright-field microscopy and FACS analysis. (B) AURKA, RHAMM, and TPX2 show cell-cycle-regulated expression in whole-cell extracts (WCEs; antibodies are those used in the rest of the study). (C) Examination of post-immunoprecipitation fractions confirmed the efficiency of the assays. Lysates following immunoprecipitation were loaded equivalently and analyzed by immunoblot to determine efficacy. (D) Protein complexes are dynamic during the cell cycle. Lysates following immunoprecipitation were loaded and analyzed by immunoblot. Arrows indicate RHAMM species with retarded mobility potentially indicative of phosphorylation. (E) As described above for AURKA, RHAMM, and TPX2, pT703-RHAMM shows cell-cycle-regulated expression in WCEs. Lysates following immunoprecipitation were loaded and analyzed by immunoblot with anti-pT703-RHAMM. Arrows show mobility consistent with pT703-RHAMM; the mobility of lower bands in AURKA, BRCA1, and TPX2 immunoprecipitations is consistent with total RHAMM.
(TIF)

Figure S9 Evaluation of pT703-RHAMM polyclonal antibody. (A) Specificity of pT703-RHAMM antibody was confirmed by immunoblot analysis. Inoculating peptide is shown. Lysates from HeLa were loaded equivalently and probed with a positive control anti-RHAMM antibody (originally named anti-IHABP). Prebleeds and bleeds from two rabbits and affinity purified antibodies against pT703 were tested by immunoblot. (B) Specificity of pT703-RHAMM antibody was confirmed by immunoblot analysis following shRNA-*HMMR*. Lysates from HeLa were loaded equivalently. Reduction in pT703-RHAMM signal was revealed following shRNA-mediated depletion of *HMMR* expression.
(TIF)

Figure S10 AURKA phosphorylates and regulates RHAMM levels. (A) AURKA abundance determines RHAMM and pT703-RHAMM levels. MCF10A cells were grown in GF-reduced media to decrease endogenous levels of RHAMM. Lysates of MCF10A cells transfected with GST-AURKA or transduced with shRNA-*AURKA* were analyzed by immunoblot analysis and compared to untreated (non-transfected and non-transduced, respectively). Abundance of both total RHAMM and pT703-RHAMM was altered by AURKA abundance. shRNA-*AURKA* reduces RHAMM levels while GST-AURKA augments RHAMM levels, consistent with the described reduction of BRCA1-mediated ubiquitination by AURKA [52] and interplay between AURKA and BRCA1 in regulating RHAMM abundance (Figure S7). (B) AURKA inhibition decreases pT703-RHAMM and, to a lesser extent, total RHAMM. Lysates of MCF10A treated with graded concentrations of an AURKA inhibitor (see Materials and Methods) were immunoblotted for the indicated proteins.
(TIF)

Figure S11 Expression profiles in mammary epithelial cells and in early stages of breast carcinogenesis. (A) Profiles of *AURKA*, *BRCA1*, *HMMR*, and *TPX2* in nonmalignant human mammary epithelial cells (immortalized clone HMT3522 S1, left panel; nonimmortalized clone HMEC 184, right panel) across days 3, 5, and 7 (two replicates for each time point are shown) in three-dimensional cultures [59]. The graphs show results for all microarray probes of the corresponding genes and *p* values of the lineal regression analyses. (B) *HMMR* expression differences between histologically normal (HN) tissues versus patient-matched atypical ductal hyperplasia (ADH) and ductal carcinoma in situ (DCIS). The graphs show results of two microarray probes (names shown at the top) and the corresponding significance *p* values (two-sided *t* test).
(TIF)

Figure S12 Effect of concurrent BRCA1 depletion and RHAMM over-expression in polarization. (A) MCF10A cells were transduced with a shRNA targeting *BRCA1* (#34) or control (pLKO.1). Additionally, cells were transduced with, or without, a vector (pLenti6.2) driving RHAMM expression. Multiplicity of infection was kept at five for single and dual transductions. Lysates were prepared 5 d post-transduction. (B) MCF10A cells, treated as indicated, were seeded in rBM to undergo polarization. After 2 weeks culture, acini were imaged and area and shape values were scored. Values were normalized to untreated controls.
(TIF)

Table S1 Variants typed in 5q34 to produce a NPL score (affected only analysis) of 4.24 among *BRCA1* mutation carrier families.
(XLS)

Table S2 Estimates (per allele _w_HR) of modification of breast cancer risk by *HMMR* rs299290 among *BRCA1* mutation carriers.
(XLS)

Table S3 Association of rs299290-C with germline *HMMR* over-expression.
(XLS)

Table S4 Genotypes of rs299290 and ER-tumor status in *BRCA1/2* mutation carriers.
(XLS)

Table S5 shRNA sequences used in this study.
(XLS)

Text S1 Supplementary Materials and Methods.
(DOC)

Text S2 Additional acknowledgments.
(DOC)

## Abbreviations

AURKA: aurora kinase A
BARD1: BRCA1-associated RING domain 1
*BRCA1*: *breast cancer 1, early onset*
CI: confidence interval
CIMBA: Consortium of Investigators of Modifiers of *BRCA1/2*
ER: estrogen receptor
GFP: green-fluorescent protein
HR: hazard ratio
LSAB: labelled streptavidin biotin
rBM: reconstituted basement membrane
VIM: vimentin
_w_HR: weighted hazard ratio
xrhamm: *Xenopus* receptor for hyaluronan-mediated motility
