# Supplementary figures and images for: Interplay between BRCA1 and RHAMM Regulates Epithelial Apicobasal Polarization and May Influence Risk of Breast Cancer
Source: PLoS Biol. 2011 Nov 15;9(11):e1001199. doi: 10.1371/journal.pbio.1001199 (PMC3217025; doi:10.1371/journal.pbio.1001199)

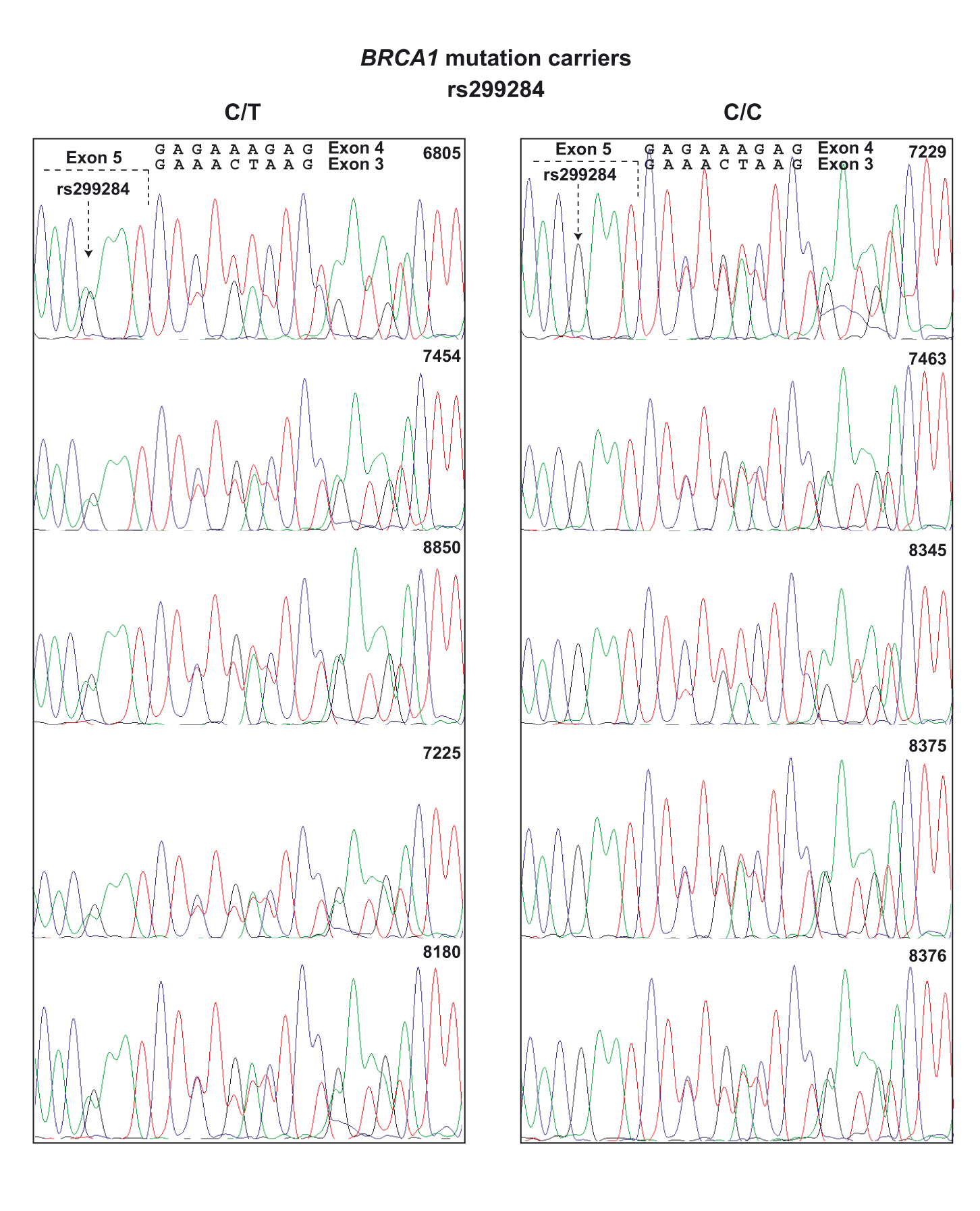

Supplement: Figure S1 — Evaluation of potential alteration of the HMMR splicing pattern by rs299284 variation. Lymphocytes from 10 BRCA1 mutation carriers were isolated and DNA and RNA samples purified for genotyping and expression analyses, respectively. Five major homozygotes and five heterozygotes for rs299284 were identified, which revealed complete linkage disequilibrium with rs299290 (unpublished data). Next, reverse transcriptase polymerase chain reactions (30 cycles) were carried out with forward (5′-GACAAAGATACTACCTTGCCTGCT-3′) and reverse (5′-CAGCATTTAGCCTTGCTTCCATC-3′) primers. Sequences were obtained using the reverse primer. Variation at rs29984 (marked by an arrow) does not alter the exon 5 acceptor donor site or the exon 4 inclusion/exclusion ratio. Sample identifiers are shown. (TIF) [file pbio.1001199.s001.tif]

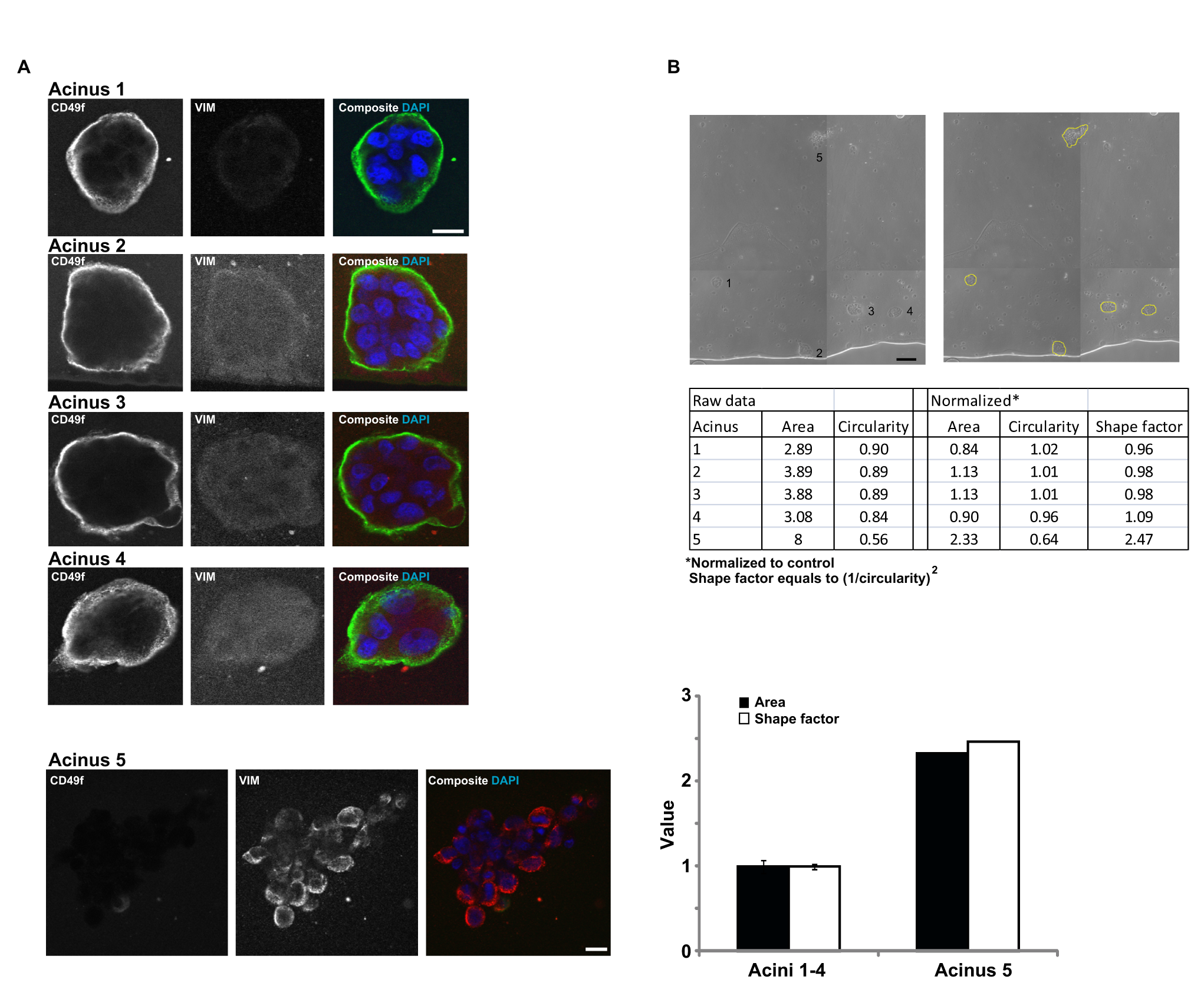

Supplement: Figure S2 — MCF10A cells grown in rBM establish apicobasal polarity and attenuate expression of VIM. (A) Apicobasal polarity and luminal characteristics were confirmed with immunofluorescence. The α6-integrin (CD49f) is deposited at the basal surface upon polarization and the expression of VIM is lost with this transition (acini #1–4). Some acini (#5) fail to polarize and do not deposit CD49f. These acini express VIM and grow larger with diminished circularity. Scale bars represent 20 µm. (B) Acini imaged with bright-field microscopy at low magnification (10×). Scale bar represents 100 µm. Quantitation of acini size and shape with ImageJ software distinguishes polarized from non-polarized acini (quantitation performed on images from 20× magnification are not shown). Normalization to controls allows comparison across replicate experiments. For display purposes, the shape factor was plotted such that values >1 indicate abnormal polarization. (TIF) [file pbio.1001199.s002.tif]

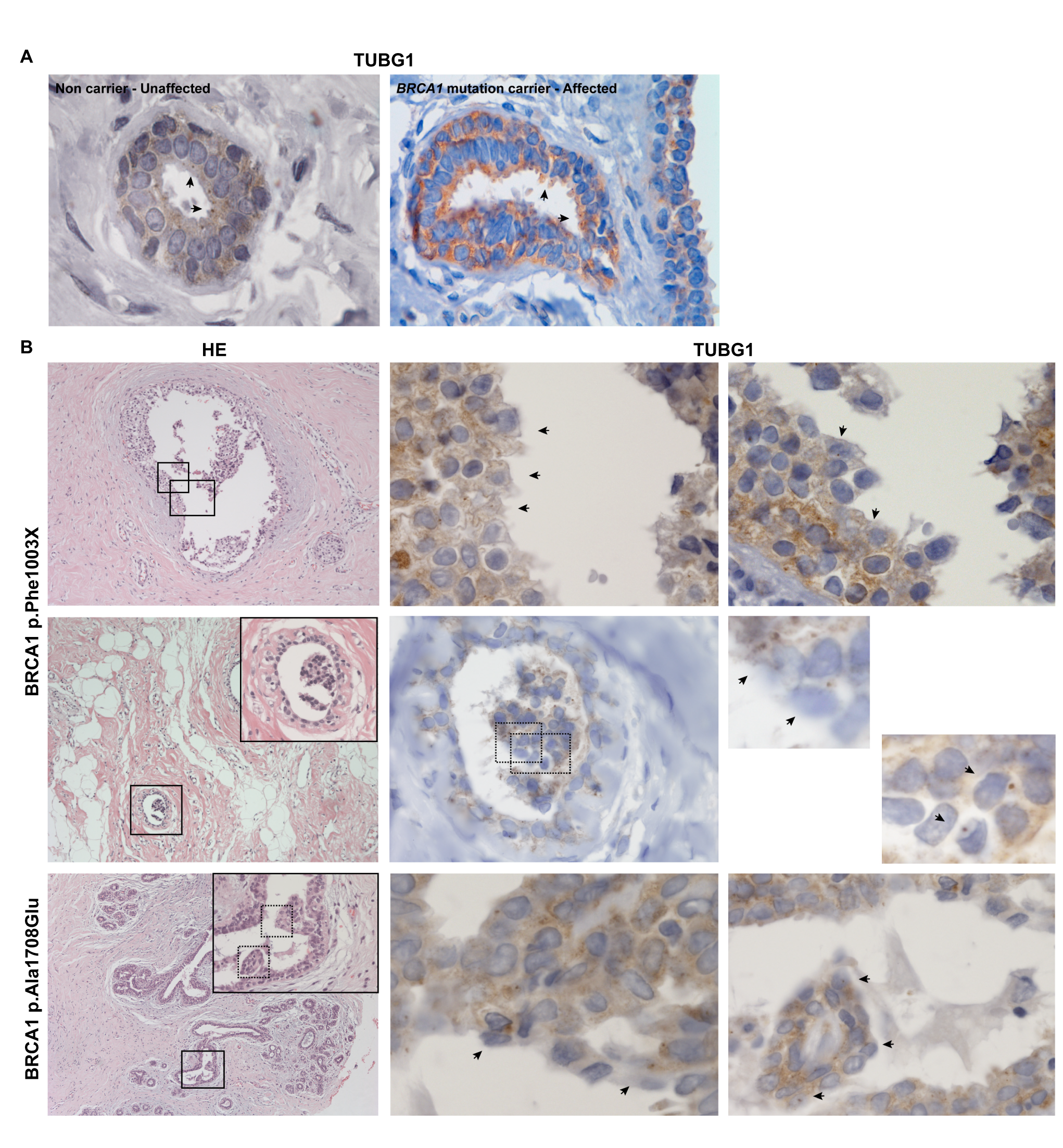

Supplement: Figure S3 — Loss of centrosome polarity in breast hyperplastic lesions of BRCA1 mutation carriers. (A) Normal luminal structure showing apical localization of the centrosomes (TUBG1) in a tissue donor (unaffected, left panel) and in a BRCA1 mutation carrier (right panel). Arrows mark properly, lumen-oriented centrosomes in many cells. (B) Three hyperplastic lesions showing loss of polarity in BRCA1 mutation carriers. Left panels show hematoxylin-eosin staining and the insets correspond to the middle and right panels with results for TUBG1 staining. Loss of polarity is evidenced by the identification of centrosome signals that are not oriented towards the lumen and/or that are located on top of the nuclei (arrows). (TIF) [file pbio.1001199.s003.tif]

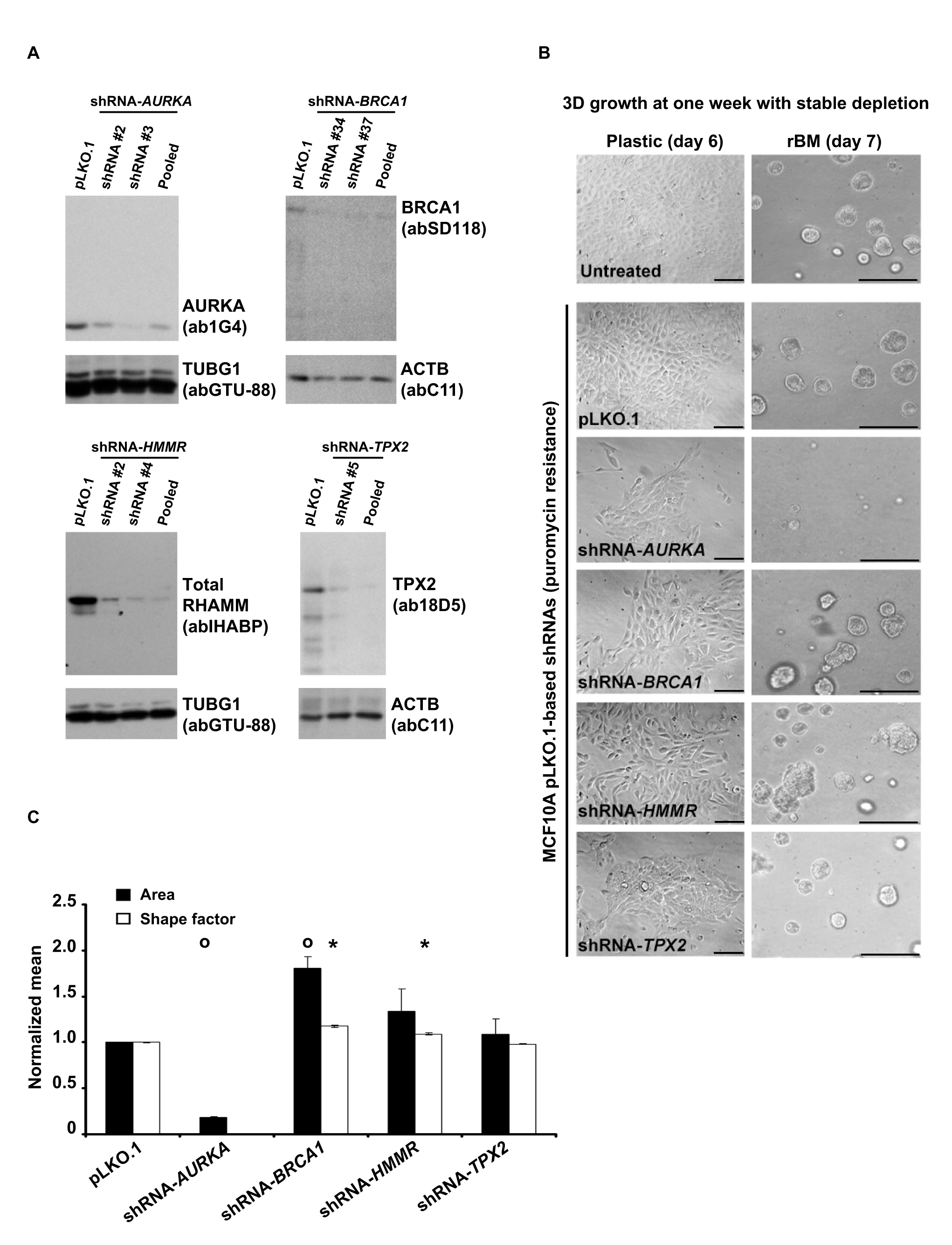

Supplement: Figure S4 — Stable depletion of centrosome components determines epithelial apicobasal polarization at an early time-point. (A) Transduction of single or pooled shRNAs targeting the expression of indicated proteins was identified resulting in detectable depletions. Sequences for the indicated shRNA are given in Table S5. (B) Representative bright-field images (low magnification, 20×) for adherent (day 6, plastic) and rBM (day 7, on-top) growth of untreated and puromycin-resistant MCF10A cells transduced with pLKO.1-nonhairpin, shRNA-AURKA, shRNA-BRCA1, shRNA-HMMR, or shRNA-TPX2. Scale bars represent 100 µm. (C) Acini architecture was quantified from bright-field images of cultures treated as indicated at 1 week post-plating. For comparison between experiments, all values were normalized to pLKO.1-transduced cultures within experiments. Shape factor ((1/circularity)2) values for single cells or small clusters are not plotted (shRNA-AURKA). Asterisks and circles indicate significant differences (two-sided t test p<0.05 and p<0.005, respectively) from controls (pLKO.1). (TIF) [file pbio.1001199.s004.tif]

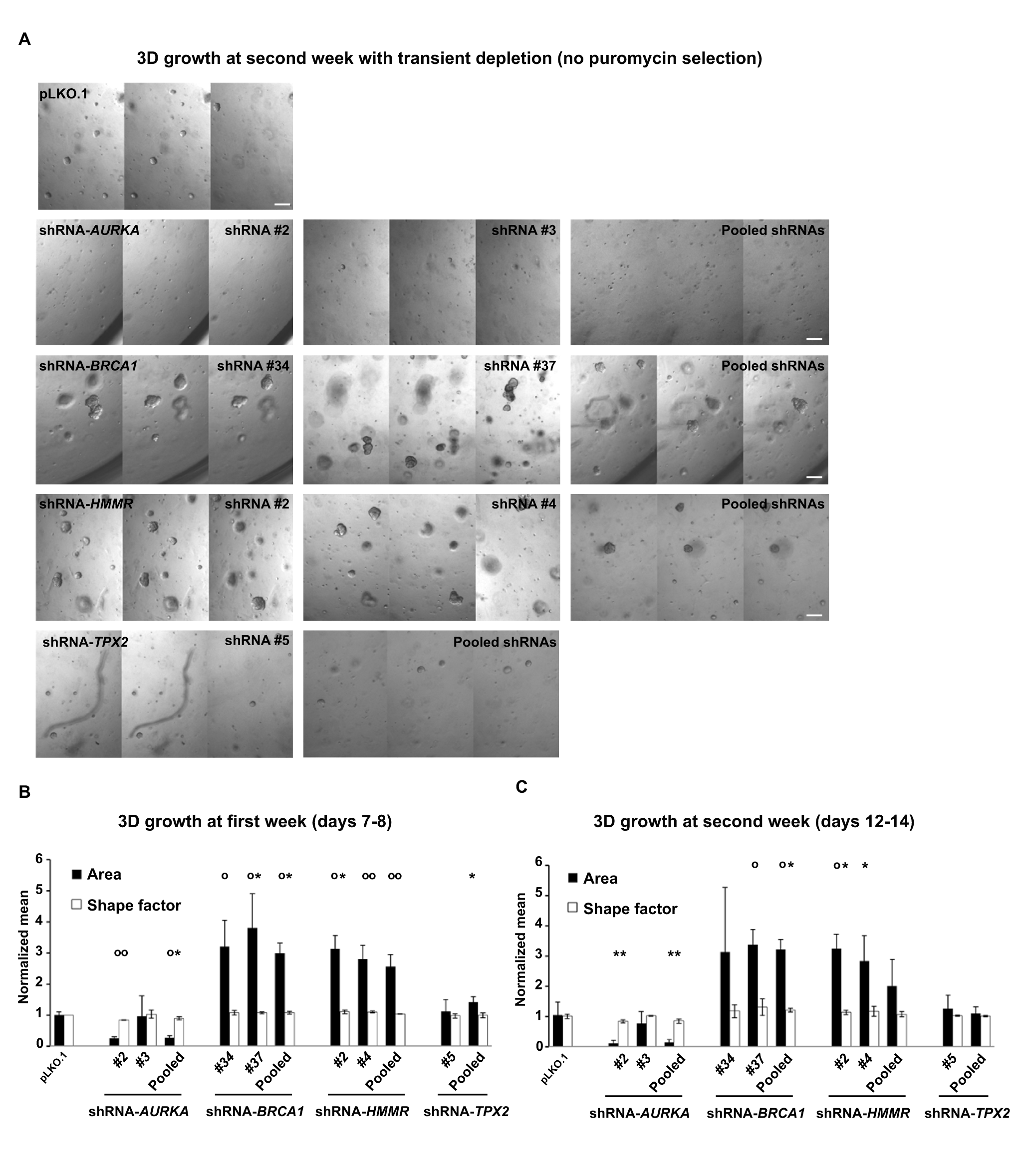

Supplement: Figure S5 — Transient depletion of centrosome components determines epithelial apicobasal polarization at early and late time-points. (A) Representative bright-field images (low magnification, 5×) for rBM (second week, embedded) growth of MCF10A transduced with virus encoding pLKO.1-nonhairpin, or individual (as indicated) or pooled shRNAs targeting the expression of AURKA, BRCA1, RHAMM, or TPX2. Images are scaled equivalently, with the scale bar representing 200 µm. (B) Acini architecture was quantified from bright-field images (10× magnification) of cultures treated as indicated at 1 week post-plating. For comparison between experiments, all values were normalized to pLKO.1-transduced cultures within experiments. Shape factor values for single cells or small clusters are not plotted (shRNA-AURKA). Asterisks and circles indicate significant differences (two-sided t test p<0.05 and p<0.005, respectively) from controls (pLKO.1). (C) Acini architecture was quantified from bright-field images (10× magnification) of cultures treated as indicated at 2 weeks post-plating. (TIF) [file pbio.1001199.s005.tif]

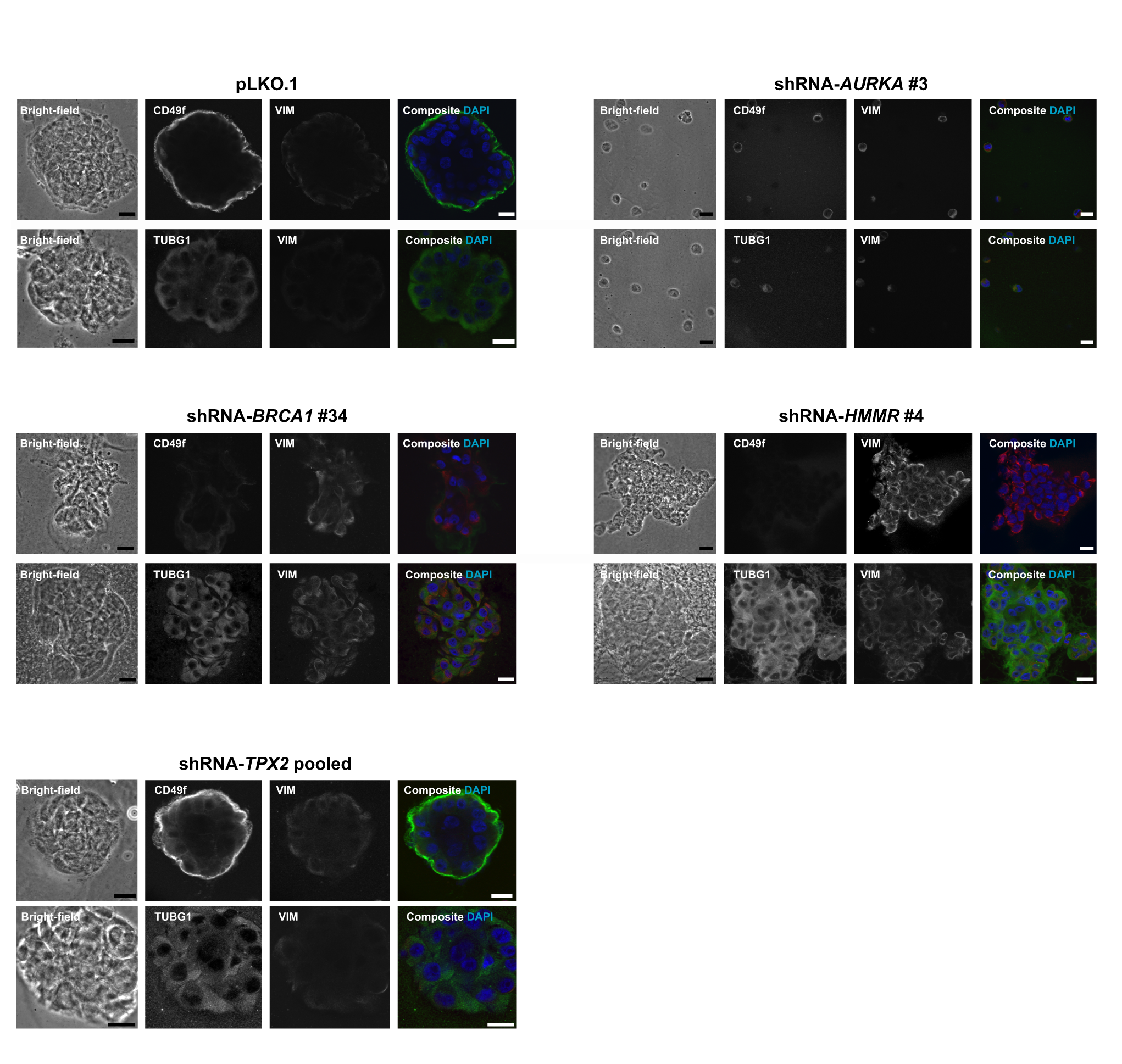

Supplement: Figure S6 — Depletion of centrosome components alters the expression profile of VIM and CD49f. In normal acini (control and shRNA-TPX2), centrosomes are apically positioned (TUBG1), CD49f is deposited at the basal surface, and VIM is lost. This pattern is altered with depletion of BRCA1 or RHAMM. Acini size and shape are reflective of polarization. Results are shown for the nonhairpin pLKO.1 control, shRNA-AURKA (#3), shRNA-BRCA1 (#34), shRNA-HMMR (#4), and shRNA-TPX2 (pooled) assays. (TIF) [file pbio.1001199.s006.tif]

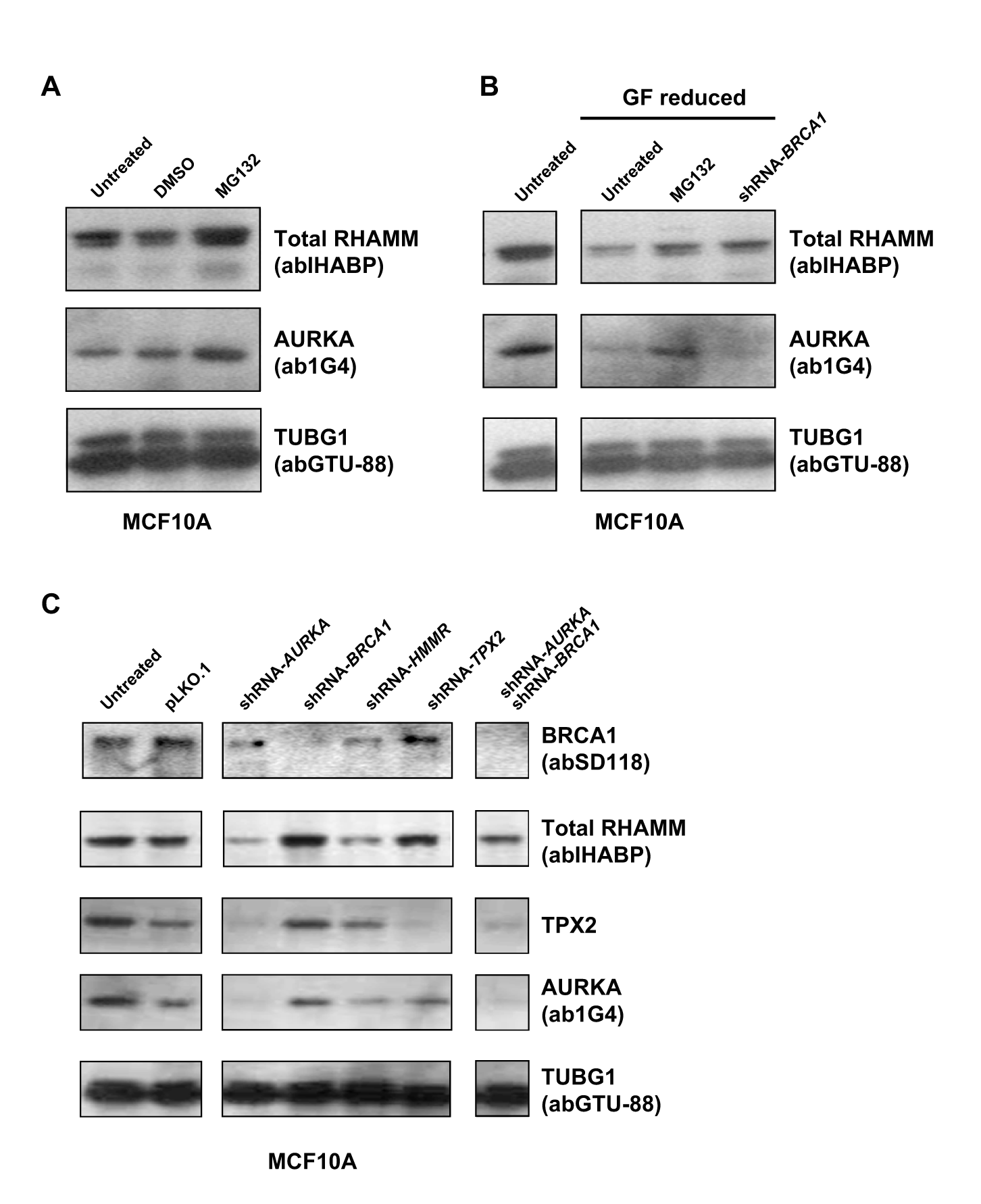

Supplement: Figure S7 — AURKA and BRCA1 activity determine RHAMM abundance. (A) AURKA and RHAMM are protected by proteasome inhibition. Lysates of MCF10A cells exposed for 3 h to DMSO or MG132 were immunoblotted for the indicated proteins. (B) BRCA1 depletion protects RHAMM, but not AURKA, from degradation. Lysates of MCF10A cells growing in growth factor (GF)-reduced media exposed to MG132 or depleted of BRCA1 were immunoblotted for the indicated proteins. (C) Interplay between AURKA and BRCA1 activity regulates RHAMM abundance. Lysates from MCF10A cultures transduced with control vector pLKO.1, shRNA-AURKA, shRNA-BRCA1, shRNA-HMMR, or shRNA-TPX2, or simultaneously with shRNAs-AURKA/BRCA1, were immunoblotted for the indicated proteins. shRNA-AURKA depletion reduces AURKA as well as RHAMM and TPX2, both cell-cycle-regulated proteins (Figure S8). shRNA-BRCA1 reduces BRCA1 and specifically increases RHAMM, which is consistent with previous data [23] and a putative role of BRCA1 in proteasome-mediated degradation of RHAMM. shRNA-HMMR and -TPX2 reduce RHAMM and TPX2 levels, respectively. Compared to single depletions, simultaneous depletion of AURKA and BRCA1 normalizes RHAMM levels but not those of TPX2. (TIF) [file pbio.1001199.s007.tif]

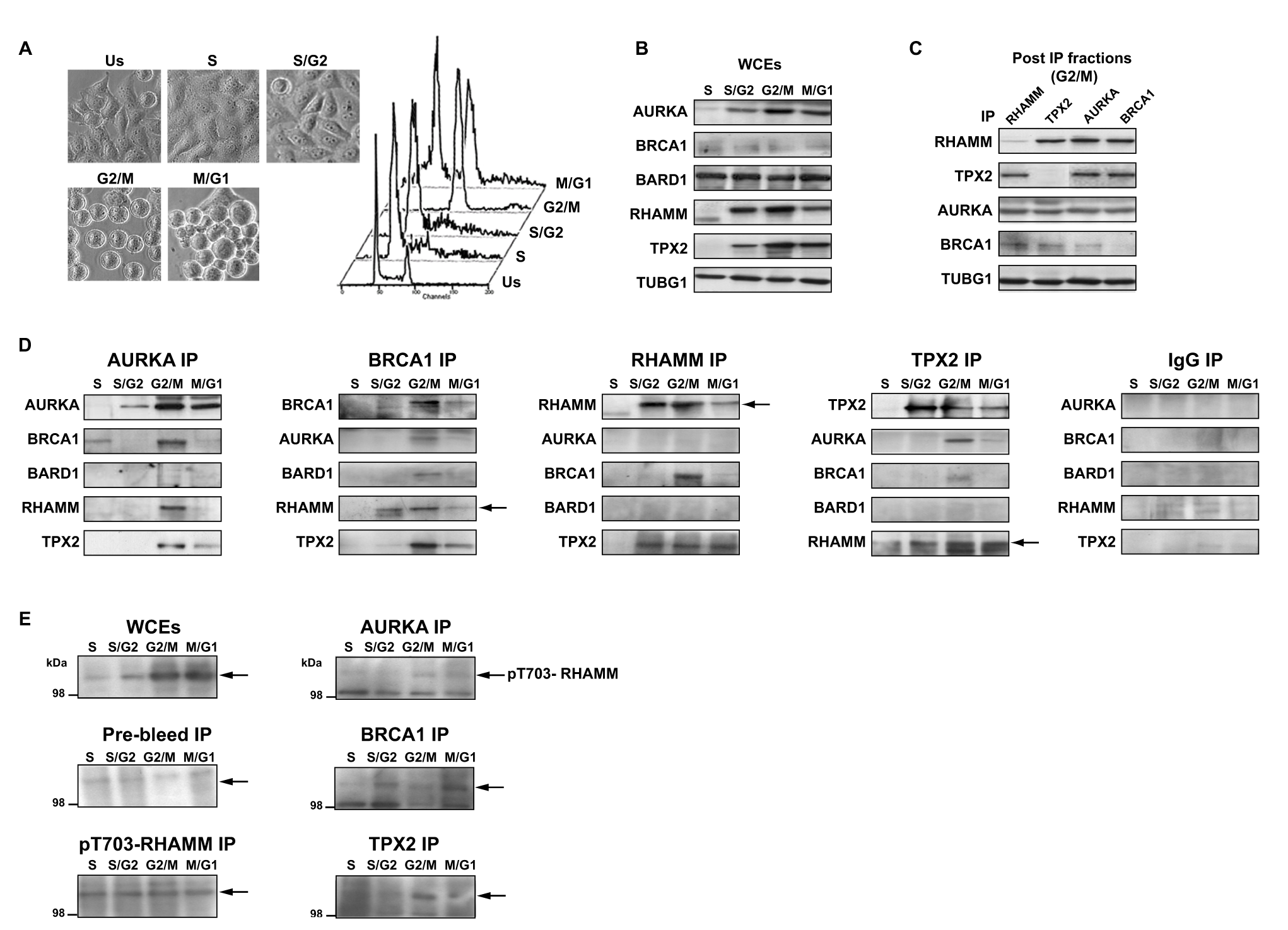

Supplement: Figure S8 — Protein complexes during the cell cycle. (A) HeLa cells were harvested at confluence (unsynchronized, Us) or following synchronization with double thymidine (S), double thymidine/nocodazole (G2/M), or release from these blocks for 3 and 5 h (S/G2 and M/G1, respectively). Synchronization was confirmed by bright-field microscopy and FACS analysis. (B) AURKA, RHAMM, and TPX2 show cell-cycle-regulated expression in whole-cell extracts (WCEs; antibodies are those used in the rest of the study). (C) Examination of post-immunoprecipitation fractions confirmed the efficiency of the assays. Lysates following immunoprecipitation were loaded equivalently and analyzed by immunoblot to determine efficacy. (D) Protein complexes are dynamic during the cell cycle. Lysates following immunoprecipitation were loaded and analyzed by immunoblot. Arrows indicate RHAMM species with retarded mobility potentially indicative of phosphorylation. (E) As described above for AURKA, RHAMM, and TPX2, pT703-RHAMM shows cell-cycle-regulated expression in WCEs. Lysates following immunoprecipitation were loaded and analyzed by immunoblot with anti-pT703-RHAMM. Arrows show mobility consistent with pT703-RHAMM; the mobility of lower bands in AURKA, BRCA1, and TPX2 immunoprecipitations is consistent with total RHAMM. (TIF) [file pbio.1001199.s008.tif]

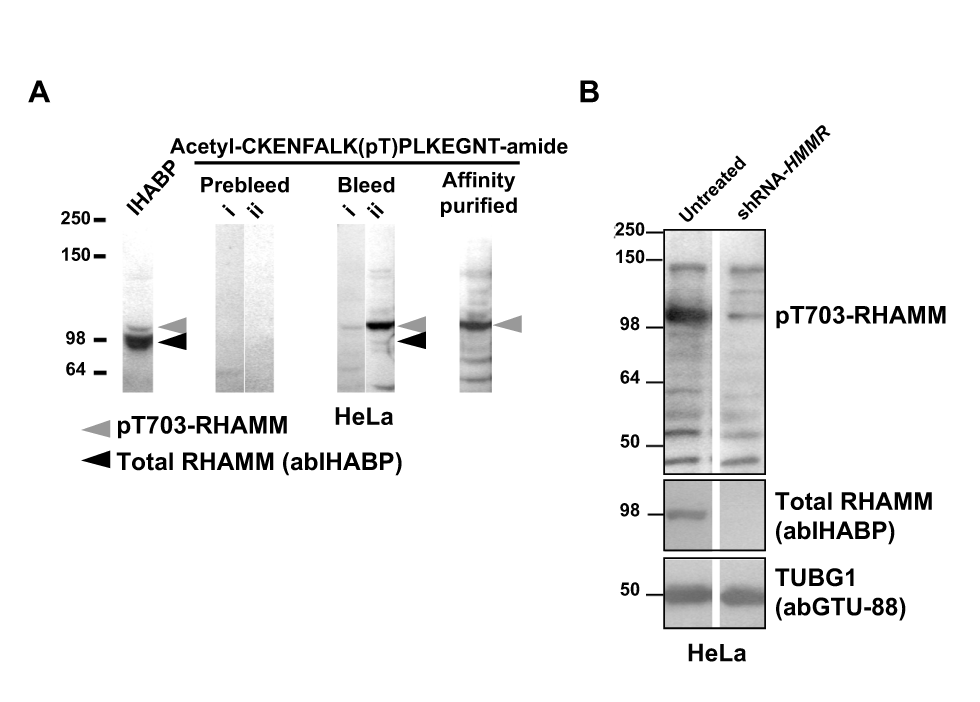

Supplement: Figure S9 — Evaluation of pT703-RHAMM polyclonal antibody. (A) Specificity of pT703-RHAMM antibody was confirmed by immunoblot analysis. Inoculating peptide is shown. Lysates from HeLa were loaded equivalently and probed with a positive control anti-RHAMM antibody (originally named anti-IHABP). Prebleeds and bleeds from two rabbits and affinity purified antibodies against pT703 were tested by immunoblot. (B) Specificity of pT703-RHAMM antibody was confirmed by immunoblot analysis following shRNA-HMMR. Lysates from HeLa were loaded equivalently. Reduction in pT703-RHAMM signal was revealed following shRNA-mediated depletion of HMMR expression. (TIF) [file pbio.1001199.s009.tif]

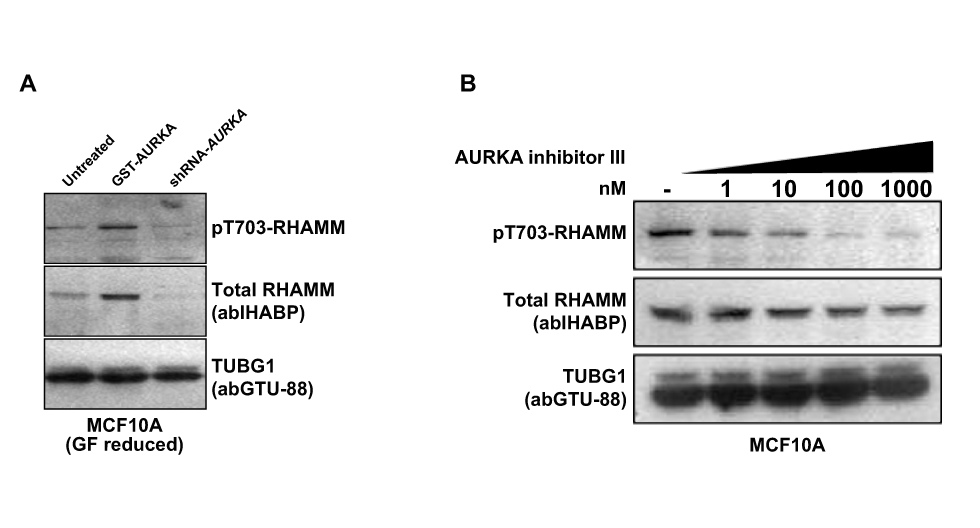

Supplement: Figure S10 — AURKA phosphorylates and regulates RHAMM levels. (A) AURKA abundance determines RHAMM and pT703-RHAMM levels. MCF10A cells were grown in GF-reduced media to decrease endogenous levels of RHAMM. Lysates of MCF10A cells transfected with GST-AURKA or transduced with shRNA-AURKA were analyzed by immunoblot analysis and compared to untreated (non-transfected and non-transduced, respectively). Abundance of both total RHAMM and pT703-RHAMM was altered by AURKA abundance. shRNA-AURKA reduces RHAMM levels while GST-AURKA augments RHAMM levels, consistent with the described reduction of BRCA1-mediated ubiquitination by AURKA [52] and interplay between AURKA and BRCA1 in regulating RHAMM abundance (Figure S7). (B) AURKA inhibition decreases pT703-RHAMM and, to a lesser extent, total RHAMM. Lysates of MCF10A treated with graded concentrations of an AURKA inhibitor (see Materials and Methods) were immunoblotted for the indicated proteins. (TIF) [file pbio.1001199.s010.tif]

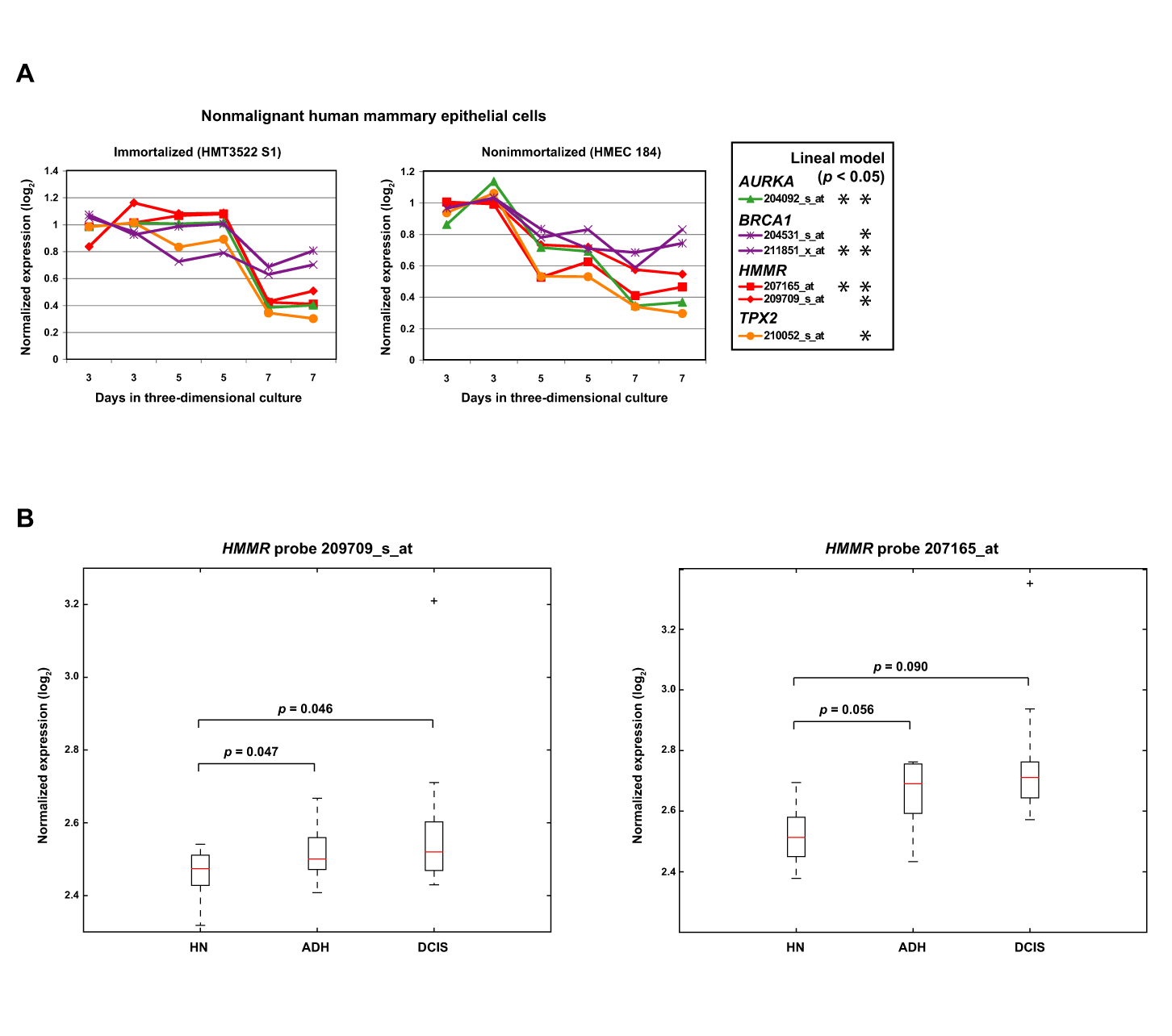

Supplement: Figure S11 — Expression profiles in mammary epithelial cells and in early stages of breast carcinogenesis. (A) Profiles of AURKA, BRCA1, HMMR, and TPX2 in nonmalignant human mammary epithelial cells (immortalized clone HMT3522 S1, left panel; nonimmortalized clone HMEC 184, right panel) across days 3, 5, and 7 (two replicates for each time point are shown) in three-dimensional cultures [59]. The graphs show results for all microarray probes of the corresponding genes and p values of the lineal regression analyses. (B) HMMR expression differences between histologically normal (HN) tissues versus patient-matched atypical ductal hyperplasia (ADH) and ductal carcinoma in situ (DCIS). The graphs show results of two microarray probes (names shown at the top) and the corresponding significance p values (two-sided t test). (TIF) [file pbio.1001199.s011.tif]

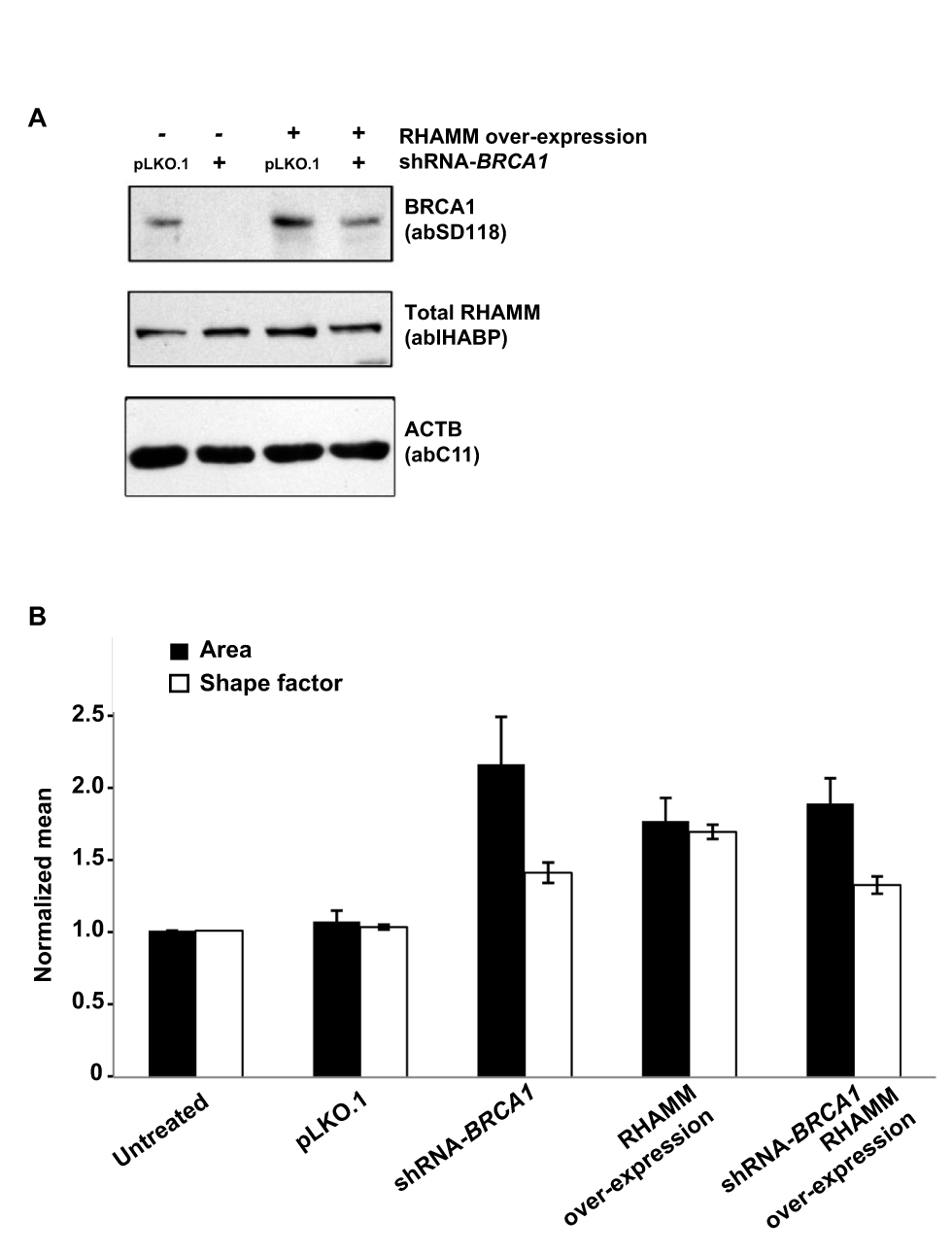

Supplement: Figure S12 — Effect of concurrent BRCA1 depletion and RHAMM over-expression in polarization. (A) MCF10A cells were transduced with a shRNA targeting BRCA1 (#34) or control (pLKO.1). Additionally, cells were transduced with, or without, a vector (pLenti6.2) driving RHAMM expression. Multiplicity of infection was kept at five for single and dual transductions. Lysates were prepared 5 d post-transduction. (B) MCF10A cells, treated as indicated, were seeded in rBM to undergo polarization. After 2 weeks culture, acini were imaged and area and shape values were scored. Values were normalized to untreated controls. (TIF) [file pbio.1001199.s012.tif]
