# Supplementary material for: Interplay between BRCA1 and RHAMM Regulates Epithelial Apicobasal Polarization and May Influence Risk of Breast Cancer
Source: PLoS Biol. 2011 Nov 15;9(11):e1001199. doi: 10.1371/journal.pbio.1001199 (PMC3217025; doi:10.1371/journal.pbio.1001199)
Supplement: Text S1 — Supplementary Materials and Methods. (DOC) [file pbio.1001199.s018.doc]

**Text S1**

**Supplementary Materials and** **Methods for**

**Interplay between BRCA1 and RHAMM Regulates Apicobasal Epithelial Polarization and May Influence Risk of Breast Cancer**

**Study Samples, Genotyping and Statistical Analysis**

Participants were enrolled if they carried recognized pathological *BRCA1* or *BRCA2* mutations (Breast Cancer Information Core criteria) and were at least 18 years old. Mutational, clinical and pathological (when available) information was provided by each contributing center (acronyms are defined in Table S2). The following individual and clinical data were collected: year of birth, mutation description, ethnicity, country of residence, age at last follow-up, age at diagnosis of breast or ovarian cancer, age at bilateral prophylactic mastectomy, age at bilateral prophylactic oophorectomy, and status and age at menopause. Initially, Italian carriers from three centers participating in the CONSIT TEAM (Centro di Riferimento Oncologico, Istituto Europeo di Oncologia, and Istituto Nazionale Tumori) and Spanish carriers (from CNIO, HSP and ICO) were genotyped at the Istituto FIRC di Oncologia Molecolare and CNIO, respectively, using 5´-3’ nuclease-based assays (TaqMan, Applied Biosystems) and following quality controls: at least 2% of duplicate samples with at least 98% concordance of genotyping results; duplicates present in each plate; random mixture of affected and unaffected carriers in a single plate; and call rate over 95% for different SNPs (when analyzed) in a single sample. All other samples were also genotyped at the corresponding centers, except for an iPLEX assay carried out at the Queensland Institute of Medical Research and containing EMBRACE, FCCC, GEORGETOWN, HEBCS, HEBON, ILUH, kConFab, MAYO, PBCS, SWE-BRCA and UPENN carriers. GC-HBOC carriers were genotyped using TaqMan assays and iCycler (Bio-Rad Laboratories). ER tumor status data were provided by groups in Iceland (ILUH), Israel (NICCC), Italy (Centro di Riferimento Oncologico, Istituto Europeo di Oncologia, and Istituto Nazionale Tumori) and Spain (CNIO, HSP and ICO).

**Cell Culture**

At day 3-5 following transfection or transduction, MCF10A were collected, counted and plated in rBM and on plastic. Cultures on plastic were grown for an additional two days, and immunoblot analysis was performed to measure the transduction or transfection, depletion or overexpression efficiencies. rBM coats (30 μl for 96 well; 100 μl for eight-well slide), plating volumes (30 μl for 96 well; 50 μl for eight-well slide) and cell concentrations (0.2 x 10^6^ cells/ml embedded; 1,000 cells/well on-top) were optimized. To analyze the effect of proteasome inhibition on polarization, MCF10A were embedded in rBM; for other endpoints, embedded and on-top conditions were equivalent. Media were changed every two days and polarization was determined two weeks post-plating. MCF10A were transduced with a lentivirus-based vector for TUBG1-GFP expression and blasticidin resistance. One week after GFP sorting, cells were selected with 5 ug/ml blasticidin for 10 days and maintained in 1 ug/ml blasticidin. To determine the effect of proteasome inhibition on polarization, MG132 (100 nM; Sigma-Aldrich) or an equivalent volume of DMSO were added to media at seeding, or as indicated, for two days. MG132 exposure was titred from 3 μM to 100 nM and from 48 hours (h) to 14 days exposure in embedded rBM cultures. For quantization of proteolysis protection, MG132 (1.5 μM) was added to adherent cultures for 3 hours (h) prior to lysis. To determine the effect of AURKA inhibition, a commercially available AURKA inhibitor (C1368; Sigma-Aldrich) was titred from 0 to 1 μM for reduction of pT703-RHAMM. The C1368 compound at 100 nM resulted in significant reduction of pT703-RHAMM, and this concentration is lower than published IC_50_ for 54 other kinases (IC_50_ for AURKA: 42 nM; IC_50_ for 54 other kinases: 131 nM for LCK, 386 nM for BMX, 591 nM for IGF1R and micromolar or higher for all other kinases tested [1,2]).

**Biochemical Assays**

For sequential immunoprecipitation, HeLa cells were synchronized using a double thymidine (2 mM) or a thymidine-nocodazole (300 ng/ml) block. Following synchronization, populations (S/G2 and M/G1) were washed three times with PBS and released into fresh media for 3 h. After defined time-points, cells were released from plates with trypsin, washed three times with PBS, and lysed at 0.5 - 1 x 10^7^ cells/ml in 150 mM NaCl lysis buffer plus protease inhibitors (Complete Protease Inhibitor Cocktail Tablets, Roche) and 50 mM NaF. Lysates were precleared with protein-A sepharose beads (GE Healthcare), incubated with antibodies (3 μg/ml of lysate) for 2 h to overnight at 4°C with rotation, and then with protein-A beads for 1 h at 4°C with rotation. Incubations for *in vitro* kinase analysis were 2 h and 1 h, respectively. Beads were collected by centrifugation and washed four times with lysis buffer. For *in vitro* kinase experiments, beads were divided in half for fast freezing (kinase assay) and SDS-PAGE analysis. Immunoprecipitated proteins were eluted with boiling SDS buffer and analyzed by a 5% stacking and 8% separating SDS-PAGE. Precleared lysates (25 μl) and post-immunoprecipitation fractions were analyzed to determine the efficiency and relative quantity of the immunoprecipitations. For immunofluorescence, cells were fixed and permeabilized in ice-cold methanol at defined time-points. Acini and/or cells were washed with PBS-0.5% Triton X-100 (Sigma-Aldrich) and blocked in skimmed milk powder (blocking buffer) before immunofluorescence. Primary and secondary antibodies were diluted in PBS-0.1% Tween (Sigma-Aldrich) and blocking buffer, and all antibodies were incubated for at least 1 h at 4°C. For double-staining experiments, antibodies were added sequentially. Cells were washed three times in PBS-0.5% Tween before and after incubations. Cells were mounted in 90% glycerol/PBS and counterstained with DAPI or TOPRO. *In vitro* phosphorylation with recombinant HIS-AURKA (PTP055, Cell Science) followed the protocol for the PKLight® HTS Protein Kinase Assay Kit (Lonza), as suggested by the manufacturer. Briefly, tests were carried out in 50 μl kinase assay buffer (50 mM HEPES pH 7.4, 3 mM MgCl_2_, 3 mM MnCl_2_, 1 mM DTT and 3 µM Na_3_VO_4_) with 4 µM ATP, 12 µM substrate (substrate A, acetyl-CKENFALK(PO_4_-T)PLKEGNT-amide; substrate B, acetyl-CKENFALK(T)PLKEGNT-amide) and 20 ng HIS-AURKA. Reactions were stopped following incubation at 30°C for indicated times. A standard curve for ATP (0-8 µM) was performed for all experiments. Reactions were performed in triplicate. Luminescence values for substrate A or substrate B were normalized to the mean value for no-substrate (HIS-AURKA alone) reactions (*n* = 12). The activity of endogenous AURKA was determined by performing the kinase assay in the absence of HIS-AURKA and the presence of ATP (4 µM), substrate B (12 µM) and beads following immunoprecipitation with IgG (negative control), anti-AURKA (positive control) or anti-TPX2 from MCF10A lysates as indicated. In untreated lysates, beads from AURKA IP, but not from TPX2 IP, contained detectable kinase activity, which was reversible through shRNA-*AURKA* depletion ([ATP]: 1.02 fold of IgG control), confirming the specificity of the assay. Luminescence values were normalized to the mean value for IgG-precipitated beads (*n* = 11). Experiments were performed in triplicate. Consumption of ATP was determined after incubation for 30 minutes.

**Gene Expression Analyses**

Raw expression microarray data of breast cancer progression [3] was downloaded from the Gene Expression Omnibus reference GSE16873 and normalized with robust multiarray average (RMA) [4], and significance analysis was performed with the significance analysis of microarray (SAM) algorithm [5]. Previously normalized and transformed data was used for the analysis of expression profiles in nonmalignant human mammary epithelial cells growing in three-dimensional cultures (GSE8096) [6]. An association study between rs299290 alleles and *HMMR* germline expression in immortalized lymphocytes of Caucasian individuals (ArrayExpress ID E-MTAB-197) [7] was performed with the SNPStats tool [8] by fitting linear equations and *p* values obtained based on the *F*-test.

**References**

1. Tari LW, Hoffman ID, Bensen DC, Hunter MJ, Nix J, et al. (2007) Structural basis for the inhibition of Aurora A kinase by a novel class of high affinity disubstituted pyrimidine inhibitors. Bioorg Med Chem Lett 17: 688-691.

2. Zhang Q, Liu Y, Gao F, Ding Q, Cho C, et al. (2006) Discovery of EGFR selective 4,6-disubstituted pyrimidines from a combinatorial kinase-directed heterocycle library. J Am Chem Soc 128: 2182-2183.

3. Emery LA, Tripathi A, King C, Kavanah M, Mendez J, et al. (2009) Early dysregulation of cell adhesion and extracellular matrix pathways in breast cancer progression. Am J Pathol 175: 1292-1302.

4. Irizarry RA, Hobbs B, Collin F, Beazer-Barclay YD, Antonellis KJ, et al. (2003) Exploration, normalization, and summaries of high density oligonucleotide array probe level data. Biostatistics 4: 249-264.

5. Tusher VG, Tibshirani R, Chu G (2001) Significance analysis of microarrays applied to the ionizing radiation response. Proc Natl Acad Sci U S A 98: 5116-5121.

6. Fournier MV, Martin KJ, Kenny PA, Xhaja K, Bosch I, et al. (2006) Gene expression signature in organized and growth-arrested mammary acini predicts good outcome in breast cancer. Cancer Res 66: 7095-7102.

7. Montgomery SB, Sammeth M, Gutierrez-Arcelus M, Lach RP, Ingle C, et al. (2010) Transcriptome genetics using second generation sequencing in a Caucasian population. Nature 464: 773-777.

8. Solé X, Guinó E, Valls J, Iniesta R, Moreno V (2006) SNPStats: a web tool for the analysis of association studies. Bioinformatics 22: 1928-1929.
