# Supplementary material for: Interplay between BRCA1 and RHAMM Regulates Epithelial Apicobasal Polarization and May Influence Risk of Breast Cancer
Source: PLoS Biol. 2011 Nov 15;9(11):e1001199. doi: 10.1371/journal.pbio.1001199 (PMC3217025; doi:10.1371/journal.pbio.1001199)
Supplement: Text S2 — Additional acknowledgments. (DOC) [file pbio.1001199.s019.doc]

**Text S2**

**Additional acknowledgements for**

**Interplay between BRCA1 and RHAMM Regulates Apicobasal Epithelial Polarization and May Influence Risk of Breast Cancer**

GEMO collaborating centers are: Coordinating Centres, Unité Mixte de Génétique Constitutionnelle des Cancers Fréquents, Centre Hospitalier Universitaire de Lyon/Centre Léon Bérard, UMR5201 CNRS, Université de Lyon (Olga Sinilnikova, Laure Barjhoux, Carole Verny-Pierre, Sophie Giraud, Mélanie Léone and Sylvie Mazoyer), and INSERM U509, Service de Génétique Oncologique, Institut Curie (Dominique Stoppa-Lyonnet, Marion Gauthier-Villars, Bruno Buecher, Claude Houdayer, Virginie Moncoutier, Muriel Belotti and Antoine de Pauw); Institut Gustave Roussy: Brigitte Bressac-de-Paillerets, Audrey Remenieras, Véronique Byrde, Olivier Caron and Gilbert Lenoir; Centre Jean Perrin: Yves-Jean Bignon and Nancy Uhrhammer; Centre Léon Bérard: Christine Lasset and Valérie Bonadona; Centre François Baclesse: Agnès Hardouin and Pascaline Berthet; Institut Paoli Calmettes: Hagay Sobol, Violaine Bourdon, Tetsuro Noguchi and François Eisinger; Groupe Hospitalier Pitié-Salpétrière: Florence Coulet, Chrystelle Colas and Florent Soubrier; CHU de Arnaud-de-Villeneuve: Isabelle Coupier and Pascal Pujol; Centre Oscar Lambret: Jean-Philippe Peyrat, Joëlle Fournier, Françoise Révillion, Philippe Vennin and Claude Adenis; Centre René Huguenin: Etienne Rouleau, Rosette Lidereau, Liliane Demange and Catherine Nogues; Centre Paul Strauss: Danièle Muller and Jean-Pierre Fricker; Institut Bergonié: Michel Longy and Nicolas Sevenet; Institut Claudius Regaud: Christine Toulas, Rosine Guimbaud, Laurence Gladieff and Viviane Feillel; CHU de Grenoble: Dominique Leroux, Hélène Dreyfus and Christine Rebischung; CHU de Dijon: Fanny Coron and Laurence Faivre; CHU de St-Etienne: Fabienne Prieur and Marine Lebrun; Hôtel Dieu Centre Hospitalier: Sandra Fert Ferrer; Centre Antoine Lacassagne: Marc Frénay; CHU de Limoges: Laurence Vénat-Bouvet; CHU de Nantes: Capucine Delnatte; CHU Bretonneau, Tours: Isabelle Mortemousque; and Creighton University, USA: Henry T. Lynch and Carrie L. Snyder. EMBRACE collaborating centers are: Coordinating Centre, Cambridge: Susan Peock, Margaret Cook, Clare Oliver and Debra Frost; North of Scotland Regional Genetics Service: Helen Gregory and Zosia Miedzybrodzka; Northern Ireland Regional Genetics Service: Patrick Morrison; West Midlands Regional Clinical Genetics Service: Trevor Cole, Carole McKeown and Laura Boyes; South West Regional Genetics Service: Alan Donaldson; East Anglian Regional Genetics Service: Joan Paterson; Medical Genetics Services for Wales: Alexandra Murray, Mark Rogers and Emma McCann; St James’s Hospital, National Centre for Medical Genetics: John Kennedy and David Barton; South East of Scotland Regional Genetics Service: Mary Porteous; Peninsula Clinical Genetics Service: Carole Brewer, Emma Kivuva, Anne Searle and Selina Goodman; West of Scotland Regional Genetics Service: Rosemarie Davidson, Victoria Murday, Nicola Bradshaw, Lesley Snadden, Mark Longmuir and Catherine Watt; South East Thames Regional Genetics Service, Guys Hospital London: Louise Izatt, Gabriella Pichert, Chris Jacobs and Caroline Langman; North West Thames Regional Genetics Service: Huw Dorkins; Leicestershire Clinical Genetics Service: Julian Barwell; Yorkshire Regional Genetics Service: Carol Chu, Tim Bishop and Julie Miller; Merseyside & Cheshire Clinical Genetics Service: Ian Ellis; Manchester Regional Genetics Service: D Gareth Evans, Fiona Lalloo and Felicity Holt; North East Thames Regional Genetics Service: Alison Male, Lucy Side and Anne Robinson; Nottingham Centre for Medical Genetics: Carol Gardiner; Northern Clinical Genetics Service: Fiona Douglas and Oonagh Claber; Oxford Regional Genetics Service: Lisa Walker and Diane McLeod; The Institute of Cancer Research and Royal Marsden NHS Foundation Trust: Ros Eeles, Susan Shanley, Nazneen Rahman, Richard Houlston, Elizabeth Bancroft, Lucia D’Mello, Elizabeth Page, Audrey Ardern-Jones and Anita Mitra; North Trent Clinical Genetics Service: Jackie Cook, Oliver Quarrell and Cathryn Bardsley; South West Thames Regional Genetics Service: Shirley Hodgson, Sheila Goff, Glen Brice and Lizzie Winchester; Wessex Clinical Genetics Service, Princess Anne Hospital: Diana Eccles, Anneke Lucassen, Gillian Crawford, Emma Tyler and Donna McBride. HEBON collaborating centers are: Coordinating Center, Netherlands Cancer Institute: Frans B. L. Hogervorst, Senno Verhoef, Martijn Verheus, Laura J. van ‘t Veer, Flora E. van Leeuwen, Matti A. Rookus; Erasmus Medical Center: Margriet Collée, Ans M.W. van den Ouweland, Agnes Jager, Maartje J. Hooning, Madeleine M.A. Tilanus-Linthorst, Caroline Seynaeve; Leiden University Medical Center: Christi J. van Asperen, Juul T. Wijnen, Maaike P. Vreeswijk, Rob A. Tollenaar, Peter Devilee; Radboud University Nijmegen Medical Center: Marjolijn J. Ligtenberg, Nicoline Hoogerbrugge; University Medical Center Utrecht: Margreet G. Ausems, Rob B. van der Luijt; Amsterdam Medical Center: Cora M. Aalfs, Theo A. van Os; VU University Medical Center: Johan J.P. Gille, Quinten Waisfisz, Hanne E.J. Meijers-Heijboer; University Hospital Maastricht: Encarna B. Gómez-García, Cees E. van Roozendaal, Marinus J. Blok; Groningen University Medical Center: Jan C. Oosterwijk, Annemarie H van der Hout, Marian J. Mourits; and The Netherlands Foundation for the Detection of Hereditary Tumours: Hans F. Vasen. SWE-BRCA collaborating centers are: Sahlgrenska University Hospital: Per Karlsson, Margareta Nordling, Annika Bergman and Zakaria Einbeigi; Linkoping University Hospital: Marie Stenmark-Askmalm and Sigrun Liedgren; Lund University Hospital: Ake Borg, Niklas Loman, Hakan Olsson, Ulf Kristoffersson, Helena Jernstrom, Katja Harbst and Karin Henriksson; Karolinska University Hospital: Annika Lindblom, Brita Arver, Anna von Wachenfeldt, Annelie Liljegren, Gisela Barbany-Bustinza and Johanna Rantala; Umea University Hospital: Beatrice Malmer, Henrik Gronberg, Eva-Lena Stattin and Monica Emanuelsson; Uppsala University Hospital: Hans Ehrencrona, Richard Rosenquist Brandell and Niklas Dahl. The kConFab study wishes to thank Heather Thorne, Eveline Niedermayr, all the research nurses and staff, the heads and staff of the Family Cancer Clinics, the Clinical Follow Up Study, and the many families who contribute. The GC-HBOC study wishes to thank Juliane Köhler for excellent technical assistance and the 12 centers of the study for providing samples and clinical data. The DKFZ study wishes to thank Diana Torres for providing samples and supplying data. The HEBCS wishes to thank Kristiina Aittomäki, Carl Blomqvist, and Hanna Jäntti for their help with patient contacts. The BFBOCC study acknowledges the Genome Database of Latvian Population, Latvian Biomedical Research and Study Centre, and Ramunas Janavicius for their contribution. The FCCC study acknowledges JoEllen Weaver, John Malick, Betsy Bove for expert technical assistance. The content of this manuscript does not necessarily reflect the views or policies of the National Cancer Institute or any of the collaborating centers in the BCFR, nor does mention of trade names, commercial products, or organizations imply endorsement by the US Government or the BCFR.
